# Sustainable Extraction of Alkaloids from *Worsleya procera*: Improving the Method with Green Chemistry

**DOI:** 10.1021/acsomega.5c08468

**Published:** 2025-11-04

**Authors:** Winner Duque Rodrigues, Ana Caroline Zanatta, Tiago Cabral Borelli, Ricardo Roberto da Silva, Carmen Lucia Cardoso, Norberto Peporine Lopes

**Affiliations:** † Research Center for Natural and Synthetic Products - School of Pharmaceutical Sciences of Ribeirão Preto, 28133 University of São Paulo, Ribeirão Preto, SP 14040-900, Brazil; ‡ Computational Chemical Biology Laboratory - School of Pharmaceutical Sciences of Ribeirão Preto, University of São Paulo, Ribeirão Preto, SP 14040-900, Brazil; § Bioaffinity Chromatography and Natural Products Group, School of Philosophy, Sciences, and Letters of Ribeirão Preto, University of São Paulo, Ribeirão Preto, SP 14040-900, Brazil

## Abstract

Green chemistry seeks to develop safer, more efficient, and environmentally sustainable processes, particularly for the extraction of bioactive compounds from natural sources. In this study, *Worsleya procera*, a chemically underexplored species of the Amaryllidaceae family, was investigated as a source of alkaloids with therapeutic potential. To the best of our knowledge, this work represents the first systematic comparison of multiple traditional and green extraction workflows applied to this species. Several protocols were evaluated using conventional solvents (methanol, hexane) and greener alternatives (ethanol, heptane), with or without modifications in the extraction steps. The optimized protocol reduced solvent use by ∼46.5% and simplified the workflow while maintaining extraction efficiency. A total of 27 alkaloids were detected, and 15 compounds belonging to the licorine, homolicorine, and tazettine subtypes were annotated. Bioaffinity chromatography revealed that extracts obtained with greener solvents showed selective inhibition of butyrylcholinesterase (76.9 ± 5.0%) compared to conventional methods (56.0 ± 6.3%), while acetylcholinesterase inhibition remained similar (26.3 ± 2.2% vs 25.7 ± 0.8%). Galantamine, used as a positive control, inhibited both enzymes by >97%, validating the bioassay. These findings demonstrate that greener extraction strategies not only reduce environmental impact but also preserve biological activity, positioning the proposed methodology as a sustainable alternative for natural product research.

## Graphical abstract

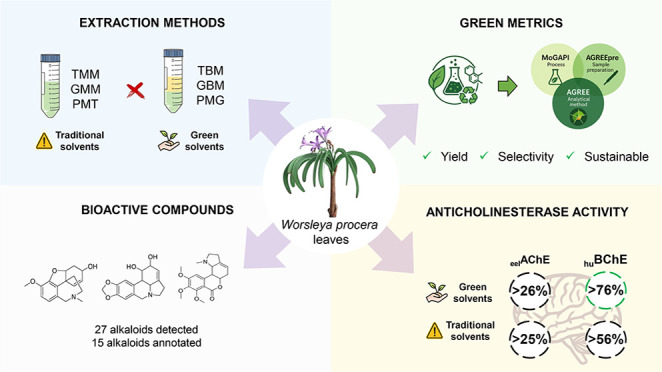

## 1 Introduction

Amaryllidaceae family represents one of the most abundant families in terms of quantity and structural diversity of alkaloids, but some species remain poorly characterized and unexplored therapeutically.^1^ Several studies have attributed various biological activities to these compounds, galantamine, for example, it was originally isolated from *Galanthus woronowii* Losinsk., is the only drug of plant origin with anticholinesterase activity used in the treatment of Alzheimer’s disease (AD).^2–5^

The therapeutic potential of plant species containing alkaloids is mainly evaluated using the polar extract obtained with methanol or hydromethanolic mixtures.^7,8^ The Stas-Otto method, developed in 1848, was adapted and became a strategy used to obtain different fractions enriched in alkaloids by liquid–liquid partitioning with organic solvents (ether, dichloromethane, chloroform, hexane) and the use of strong acids (sulfuric acid, hydrochloric acid) and bases (sodium and ammonium hydroxides) as pH modifiers.^9^ Depending on the method used, alkaloids may be obtained in free or base form. Several factors are known to affect the stability of alkaloids, including exposure to light, heat, and pH extremes.^10^

In addition, the solvents commonly used in the traditional method pose a risk to the health and safety of the analyst, laboratory equipment, and most importantly, the environment. Green Analytical Chemistry (GAC) is a field that promotes the rationalization of all processes involved in the development and research chain of bioactive compounds. This emerging field is based on 12 fundamental principles, including the use of renewable raw materials, waste reduction, the use of safe solvents, energy consumption and process efficiency. Solvents are one of the first substitutions made to minimize the impact and costs generated in the process.^11–14^

Innovative processes following the principles of GAC have been highlighted and have been shown to be relevant, especially in the last 20 years.^14^ Traditional extraction methods often rely on toxic organic solvents (e.g., methanol, hexane, dichloromethane, ether) and energy-intensive processes (e.g., factors such as temperature and pressure changes, heating, prolonged agitation, etc.), resulting in negative environmental impacts and health risks for operators. The search for more sustainable extraction methods is, therefore, a priority for research in natural products and analytical chemistry.^13^

An effective way to identify the lack of sustainability in laboratory methods is through the use of specific metrics. These metrics allow for the evaluation of the sustainability of processes from collection and processing to obtaining analytical data. Among the most widely used tools for this purpose are the Green Analytical Procedure Index (GAPI)^15^ and the Analytical GREEnness Evaluation (AGREE).^16^ Both tools can measure the impact of each method, thus facilitating the comparison between them. AGREE performs a quantitative analysis using a radar-type graph with a score ranging from 0 to 1, the higher the value, the more sustainable the method. In addition, the AGREE metric is strictly related to the principles of GAC.^17–19^

Different methodologies have been developed to improve the extraction of alkaloids and obtain enriched fractions with high yields for analytical purposes, from sample preparation to chemical characterization.^20,21^ However, studies on the extraction of alkaloids from Amaryllidaceae are scarce, and the few that exist use techniques that involve multiple steps that require specialized labor, expensive resources, such as supercritical CO_2_ and deep eutectic solvents, which are often inaccessible to small research laboratories.^22–24^

Chibli and collaborators^21^ recently demonstrated the possibility of obtaining fractions rich in different alkaloid subtypes using a simple methodology with green and easily accessible solvents, and with a good degree of reproducibility compared to traditional methods. In addition, optimized extraction techniques using smaller sample volumes, green solvents, and multiphase extraction systems can reduce analysis time and are already a reality for other classes of secondary metabolites.^8,25,26^

Nevertheless, to explore the chemodiversity of Amaryllidaceae, *Worsleya procera* was chosen due to its biological potential and chemical characterization. It is a Brazilian native species, endemic to the Serra dos Órgãos region in the state of Rio de Janeiro, which has not yet been explored. The only mention in the literature was the antiplasmodial and cytotoxic potential of the alkaloids present in its roots.^6^ However, it is known that alkaloids in Amaryllidaceae are also present in the aerial parts.^1^

The anticholinesterase (anti-ChE) potential is one of the most studied activities in the Amaryllidaceae, methods for identifying potential inhibitors use enzymatic assays in solution based on colorimetric methods with Ellman’s reagent^27^ or the Fast Blue B salt reagent.^28,29^ However, alternatives to colorimetric methods have been discussed because the accuracy of the assays depends on the nature of the sample, the lack of standardization of the assay, and possible interactions between reagents.^30–33^ In this sense, selective affinity chromatography has been extensively studied to mimic biological actions, allowing rapid evaluation of thermodynamic and kinetic constants, as well as screening and determining the mechanisms of action of inhibitors.^34,35^ For example, the anticholinesterase activity of both acetylcholinesterase and butyrylcholinesterase enzymes was determined using a bioaffinity liquid chromatography system.^36–38^

Acetylcholinesterase (AChE) and butyrylcholinesterase (BChE) are key enzymes in cholinergic neurotransmission, responsible for the hydrolysis of acetylcholine and butyrylcholine, respectively. Their dysfunction has been strongly associated with the progression of AD, making them important therapeutic targets in the search for new inhibitors. Therefore, evaluating the inhibitory activity of alkaloid-rich extracts against AChE and BChE not only supports the pharmacological relevance of these compounds but also provides insights into their potential application in the management of neurodegenerative disorders.^2–5^

In previous studies, this method was found to be fast, economical and reproducible, making it a relevant strategy for analyzing the bioactive potential of natural products.^36–38^ Therefore, the aim of this work is to compare different extraction methods, such as monophase and biphase dynamic maceration, using traditional solvents (methanol, hexane) and reagents (sulfuric acid, sodium hydroxide), which are considered toxic and unsafe, and alternative solvents (ethanol, heptane) and reagents (citric acid, ammonium hydroxide), which are safer and more environmentally friendly.^11–13^ We have developed an extraction method with fewer steps and without the use of acids while maintaining high extraction efficiency, enabling the assessment of *Worsley procera*’s anti-ChE potential.

## 2 Experimental Section

### 2.1 Chemicals and Reagents

The solvents utilized for sample extraction included methanol and ethanol (Merck), as well as heptane, hexane, and ethyl acetate (EtOAc) (Synth). Additionally, sulfuric acid (H_2_SO_4_), citric acid, sodium hydroxide (NaOH), and ammonium hydroxide (NH_4_OH) solutions were used as pH modifiers. The C8–C40 *n*-alkane standard (50 mg/L in hexane, Sigma-Aldrich) was used for retention index calibration in GC-MS analysis.

### 2.2 Plant Material

The plant material was collected in Mogi das Cruzes (São Paulo, Brazil; 23°39′55.97″ S, 46°7′53.11″ W) in November 2022 (SisGen license no. AE355E3, see Supporting Information DOC S1). Leaves from various individuals (*n* = 6) of the *Worsleya procera* species were collected and stored on ice. To stabilize the plant material, the samples were immersed in liquid nitrogen, pulverized, dehydrated using a freeze-dryer (LioTop model L101), and stored at −20 °C for further analysis.

### 2.3 Extraction

#### 2.3.1 Monophasic Extractions

For the traditional monophasic method (TMM), six steps were performed: (i) maceration with 100 mg of plant material and 30 mL MeOH:H_2_O (80:20 v/v) under stirring at 2000 rpm on a magnetic stirrer (Solab model SL-91) for 10 min without heating, followed by evaporation of the solvent with N_2_; (ii) the dried hydromethanolic extract was resuspended in water and acidified to pH ≈2.0 with 3 M H_2_SO_4_; (iii) liquid–liquid extraction with hexane was performed to remove interferents from the acidified aqueous extract; (iv) 3 M NaOH was added to the aqueous extract until pH ≈10.0 was reached; (v) liquid–liquid extraction with 10 mL of hexane (3×) and then (vi) with 10 mL of EtOAc (3×) were performed for alkaloid recovery. For comparison, a green monophasic method (GMM) was employed, following the same steps and proportions. In this method EtOH:H_2_O (70:30 v/v) was used for extract preparation, citric acid 3 M and NH_4_OH 3 M as pH modifiers, and heptane and EtOAc as organic solvents for the liquid–liquid extractions (see Supporting Information Figure S1A and Table S1).

#### 2.3.2 Biphasic Extractions

For the traditional biphasic method (TBM), the biphasic dynamic maceration was performed with a (i) 1:1 (v/v) mixture of 15 mL MeOH:H_2_O (80:20 v/v) and 15 mL hexane, while for the green biphasic method (GBM) the same ratio of EtOH:H_2_O (70:30) and heptane was used. All other steps (ii–vi) described in Section 2.3.1 were followed for each of the respective methods (see Supporting Information Figure S1B and Table S1).

#### 2.3.3 Modified Partition

For the modified partition, three steps were followed that were (i) identical to those described in Section 2.3.1, using both traditional (PMT) and green (PMG) methods. Subsequently, (ii) the addition of acids was eliminated, with only resuspension in water and addition of 3 M NaOH for modified method using traditional solvents (PMT) and 3 M NH_4_OH for modified method using alternative solvents (PMG) until pH ≈10.0 was reached; (iii) liquid–liquid extraction with 10 mL EtOAc (3×) for recovery of alkaloids in each of the methods separately (see Supporting Information Figure S2 and Table S1). All steps are outlined in Figure 1.

**Figure 1:**
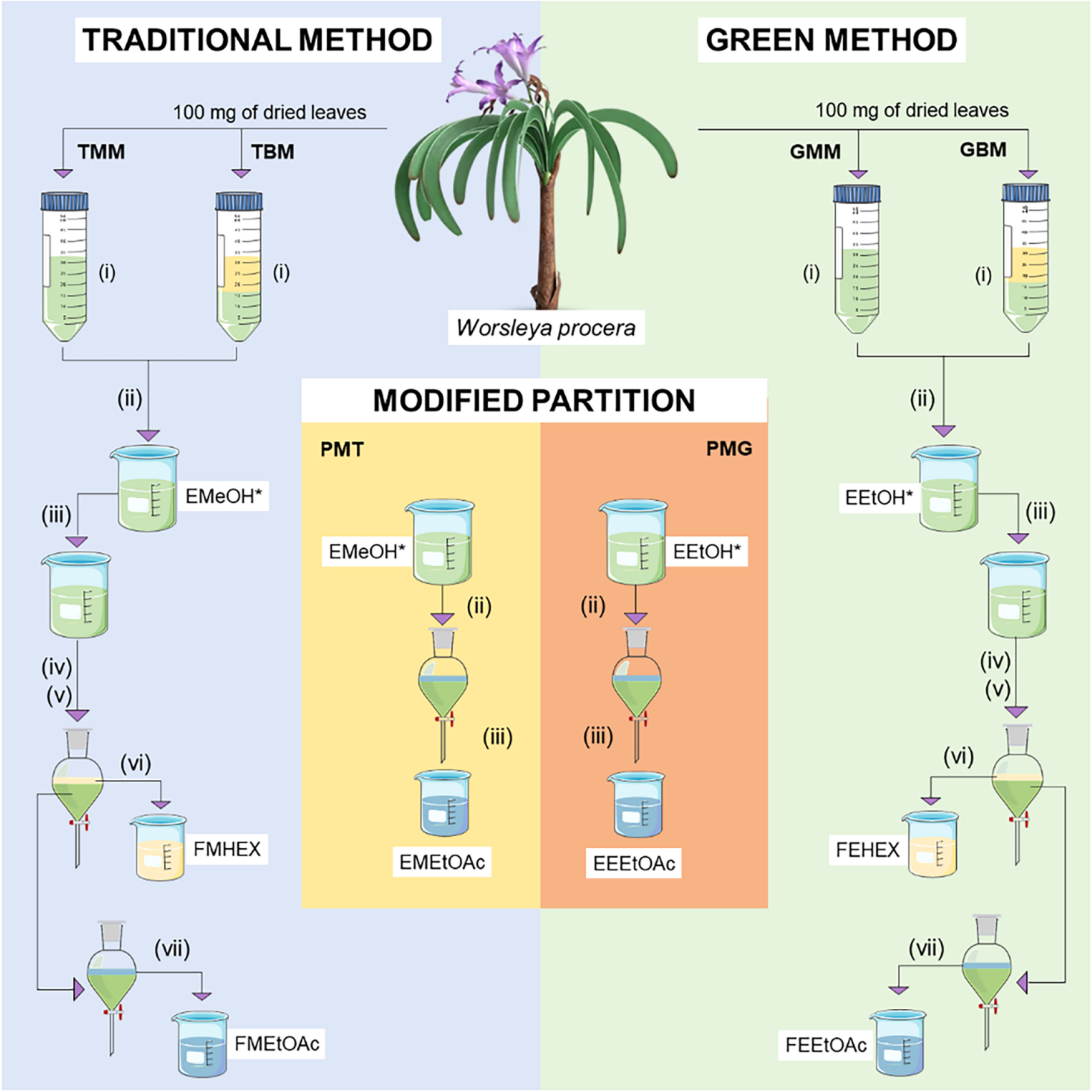
Workflow scheme for obtaining enriched alkaloid fractions of *Worsleya procera* leaves. Legend: (*) Only hydroalcoholic extracts obtained by biphasic extraction proceeded to the next stages.

### 2.4 Gas Chromatography Coupled to Mass Spectrometry Analysis

The fractions obtained from all the different extraction methods were resuspended and solubilized in high-purity EtOAc at a concentration of 10 mg/mL. The samples were filtered through a 0.22 μm hydrophobic PTFE membrane (Analitica, China). EtOAc was injected as a blank for the analysis. The spectra were obtained on a Shimadzu GCMS-QP2010 Ultra system (Tokyo, Japan), using a Zebron ZB-5MS column (30 m × 0.25 mm × 0.25 μm) (Phenomenex, Torrance). The temperature programming was as follows: starting at 100 °C with 3 min hold, temperature increase at 15 °C/min up to 180 °C, held for 1 min, and subsequently, an increase of 5 °C/min up to 310 °C, held for 20 min. The injector temperature was 260 °C, and 1 μL of sample was injected into a split injection mode (1:5). Helium was used as the carrier gas at a flow rate of 1.00 mL/min operating in electron ionization (EI) mode at 70 eV Data acquisition started at 4 min into the run with an ion source temperature of 250 °C and interface temperature of 310 °C. Full-scan MS spectra were acquired in the range of *m*/*z* 35–600, with an event time of 0.30 s and a scan speed of 2000 u/sec. At the end of the sequence, the standard *n*-alkane mixture and a solvent blank containing only ethyl acetate were analyzed. The Shimadzu GCMS-Solution software (version 4.20, Shimadzu, Tokyo, Japan) was used to acquire and process the chromatographic and spectral data.

### 2.5 Green Chemistry Metrics

The greenness of the extraction methods was assessed using three complementary tools based on the 12 principles of Green Analytical Chemistry. The modified Green Analytical Procedure Index (MoGAPI) (available at: https://fotouhmansour.github.io/MoGAPI/)^39^ was used for a visual overview, while the Analytical GREEnness Evaluation calculator (AGREE) software v. 0.4 (available at: https://mostwiedzy.pl/AGREE)^16^ and Analytical Greenness Metric for Sample Preparation (AGREEprep v. 0.91) (available at: https://mostwiedzy.pl/pl/wojciech-wojnowski,174235-1/agreeprep)^40^ tools provided quantitative scores for the overall procedure and sample preparation, respectively.

To more accurately reflect the methodological changes from the traditional method to the green method, a weight of 3 was assigned to the principles directly affected by the changes made (e.g., choice of solvents, number of steps and reagent safety), while the unchanged principles retained a standard weight of 1 (see Supporting Information Table S1).

### 2.6 Acetylcholinesterase and Butyrylcholinesterase Inhibition Screening Assays

The inhibition screening assays were carried out using an immobilized capillary enzyme reactor (ICER) based on acetylcholinesterase from *Electrophorus electricus* (_eel_AChE, Type VI–S; EC 3.1.1.7) and human serum butyrylcholinesterase (_hu_BChE, EC 3.1.1.8) referred to _eel_AChE-ICER and _hu_BChE-ICER, respectively. The enzymes were covalently immobilized independently onto fused silica capillary (30 cm x 0.375 mm x 100 μm i.d.) as described by Seidl et al.^37^ Both _eel_AChE-ICER and _hu_BChE-ICER were individually connected to an LC-IT-MS/MS system. The LC system (Nexera XR, Shimadzu) comprised two LC-20AD pumps, a SIL20A autosampler with a 50 μL loop, a DGU-20A5 degasser and a CBM-20A interface. The LC system was coupled to an AmaZon Speed Ion Trap (IT) mass spectrometer (MS) equipped with an ESI source and an ion trap analyzer controlled by Compass 1.7 software (Bruker Daltonics). The MS operated in a positive mode (scan *m*/*z* 50–250). IT-MS parameters were configurated as follows: capillary voltage = 4.500 V, end plate voltage= 500 V, drying gas flow rate 6.0 L/min, drying temperature = 280 °C, and nebulizer pressure = 30 psi. Nitrogen and helium were used as sheath gas and collision gas, respectively. Data acquisition was carried out using Bruker Data Analysis Software (version 4.3).

The enzymatic reaction was monitored by directly quantifying the enzymatic hydrolysis product at *m*/*z* 104 using galantamine as a standard inhibitor in accordance with published procedure.^36–38^ All analyses were conducted at room temperature (21 °C). Stock sample solutions were prepared in MeOH at a concentration of 2 mg/mL and centrifuged at 10,000 rpm for 5 min. The running buffer consisted of an ammonium acetate solution (15 mM, pH 8.0) was used at flow rate of 0.05 mL/min. Reaction mixtures (100 mL; final concentration 200 μg/mL) were prepared by combining 10 μL of the sample solution (2 mg/mL), 20 μL of acetylcholine (70 μM) for _eel_AChE or 20 mL of butyrylcholine for _hu_BChE and 70 μL ammonium acetate buffer (15 mM, pH 8.0). A 10 μL aliquot of each reaction mixture was injected in triplicate into the LC-MS system and the inhibition percentages were calculated according to eq 1. by comparing the product peak area in the presence (P_i_) and in the absence (P_0_) of the inhibitor, subtracting the area of choline resulting from the spontaneous substrate hydrolysis. For this control, an empty capillary was used. Positive controls were conducted without an inhibitor, while negative controls assessed sample interference with substrate autohydrolysis.

1 $$\%I=100-\left[\left(P_{i}/P_{0}\right)\;\times \;100\right]$$

### 2.7 Spectral Data Processing

The raw data obtained by GC–MS were converted to the open format .*mzXML* using the software MSConvert (ProteoWizard, v3.0) with the peak picking filter enabled. The converted files were processed in MZmine v4.4.3 ^41^ following a standardized workflow. Initially, mass detection was performed for all scan types using the Mass Detection module, with a noise level threshold of 2.0 × 10^1^. Chromatograms were then built with the ADAP Chromatogram Builder using a minimum of 4 consecutive scans above an intensity threshold of 1.0 × 10^4^ and a minimum peak height of 2.0 × 10^4^, without allowing single-scan chromatograms. Peak deconvolution was carried out with the Minimum Search feature resolver, applying a chromatographic threshold of 0.8, a minimum search range of 0.05 min, a peak top/edge ratio ≥1.8, a minimum absolute height of 1.0 × 10^4^, and at least 3 scans per peak. Spectral deconvolution was further refined using the Spectral Deconvolution GC module to resolve overlapping spectra. The Rows Filter module was applied to retain only features meeting isotope pattern criteria (minimum of 2 isotopic peaks), while preserving MS/MS and unannotated features. Feature alignment across samples was achieved with the GC Aligner using a retention time tolerance of 0.1 min and a weight for retention time of 0.5. The resulting feature quantification table was exported in.csv format for subsequent PCA and UpSet data analysis.

### 2.8 Statistical Analysis

All experiments with the six collected individuals were conducted in triplicate biological replicates (*n* = 3). Data normality and homogeneity of variances were verified prior to statistical testing. Comparisons between groups were conducted using one-way analysis of variance (ANOVA), followed by Bonferroni’s multiple comparison post hoc test to identify significant differences among treatments. The results are expressed as mean ± standard error of the mean (SEM). A significance level of *p* < 0.05 was adopted. Statistical analyses were carried out using GraphPad Prism version 9.

Principal component analysis (PCA) was performed in Python (version 3.12) using GC-MS data processed according to item 2.7. Peak areas were normalized to total ion count per sample and autoscaled (mean-centered and scaled to unit variance). PCA was computed with the scikit-learn library, and 95% confidence ellipses were plotted based on the first two principal components. The analysis included the samples EMeOH, FMHEX, FMEtOAc, EMEtOAc, EEtOH, FEHEP, FEEEtOAc, and EEEtOAc. The code used for data processing and visualization is available at: https://github.com/computational-chemical-biology/Worsleya-procera-data-and-code.

## 3 Results and Discussion

### 3.1 Extraction of Samples

The development of extraction methods for *W. procera* leaves was driven by the need for efficient alkaloid extraction, environmental and safety concerns, and economic considerations. Various factors, including solvent compatibility with target compounds, solvent ratio, stirring, and temperature, influence extraction efficiency. Considering this, we compared the steps taken in utilizing both monophase and biphasic extraction systems for optimal alkaloid recovery.

To enhance the selectivity of subsequent extractions, we implemented a preliminary cleanup stage using nonpolar solvents (hexane and heptane). This step was used to remove low-polarity compounds such as hydrocarbons, phytosterols, and fatty acids, which could interfere with the extraction of alkaloids. By eliminating these interfering substances early on, we ensured that subsequent extraction steps were more efficient and targeted toward alkaloids, which are relatively polar and have higher solubility in polar or moderately polar solvents.^9,42^

In the monophase system (see Supporting Information Figure S1A), plant material was immersed in a single extraction solvent and stirred for a specific period, with each solvent used separately for extraction. This method allows for sequential extraction of compounds based on their polarity. In contrast, the biphasic system (see Supporting Information Figure S1B) involved immersing the plant material in a mixture of solvents that, when agitated, simultaneously extracted substances according to their polarity.^43–45^ This approach reduced extraction time by obtaining both apolar and polar extracts simultaneously from a single sample, aligning with Green Analytical Chemistry (GAC) principles.^12,13^

Figure 2A shows that, within the same extraction system, the yield was not significantly affected by the solvent type, whether traditional (hexane, hydromethanol) or classified as “green” (heptane, hydroethanol). The choice of ethanol:H_2_O (70:30 v/v) was based on its polarity, which is comparable to methanol:H_2_O (80:20 v/v), a mixture commonly employed for obtaining polar extracts from Amaryllidaceae species. This suggests that greener solvent alternatives can be implemented without compromising extraction efficiency, provided the overall extraction strategy remains constant. The chemical profiles obtained from both extraction methods were similar (see Supporting Information Figures S3 and S4), suggesting that while the biphasic method is efficient, even with lower yield (Figure 2B). The data obtained for the biphasic system are consistent with previous studies using similar approaches.^44,46^

**Figure 2:**
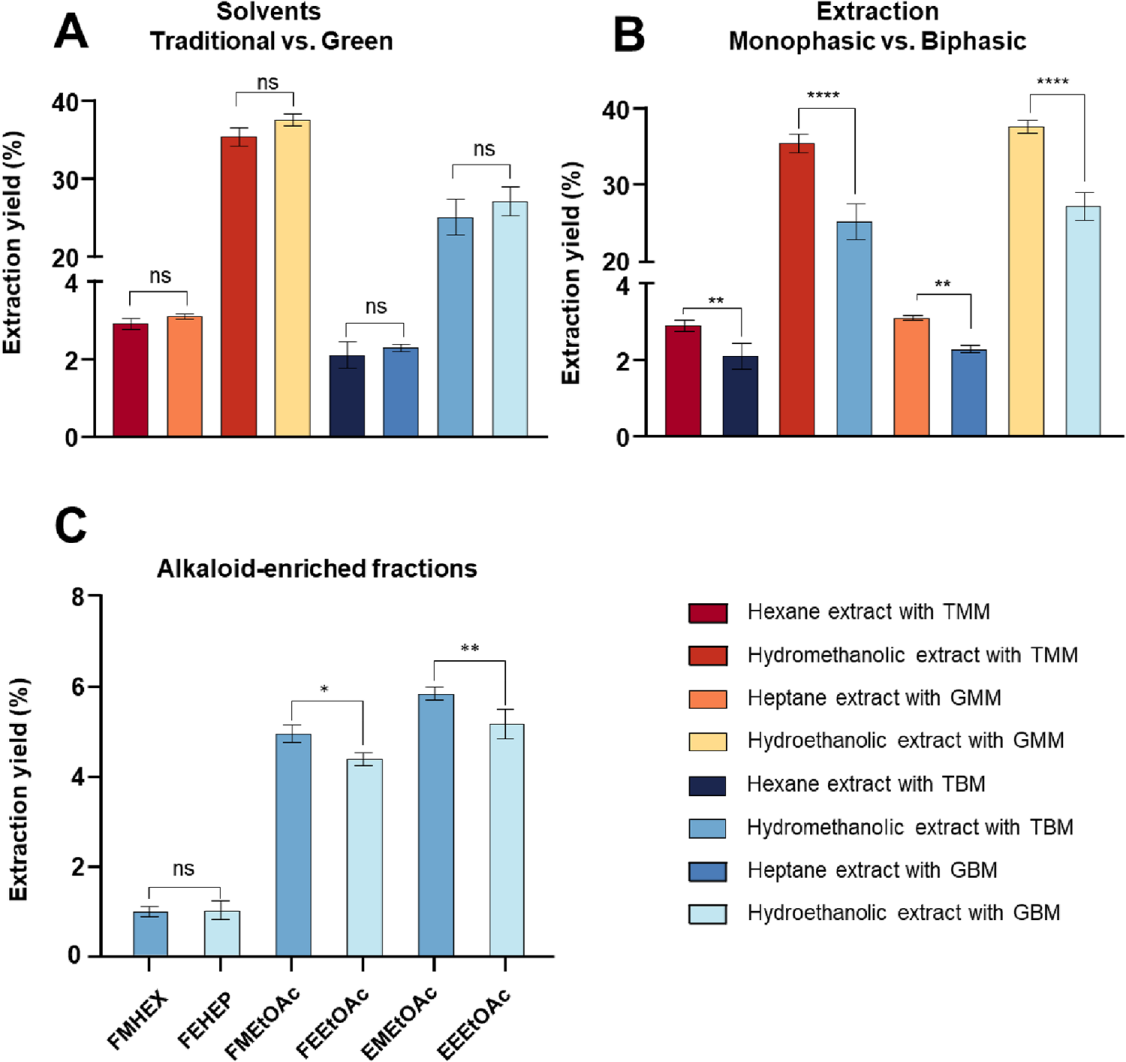
Extraction yield (%) of *Worsleya procera* leaves using different methods and solvents. Legend: (A) Comparison between traditional solvents and green solvents. (B) Comparison between monophasic and biphasic extraction methods. (C) Comparison between the yield of the alkaloid-enriched fraction. Yields are expressed as mean ± standard error of the mean (*n* = 3). Statistical differences were evaluated by one-way ANOVA followed by Bonferroni’s multiple comparison test. ns = not significant, (*p* > 0.05),* *p* < 0.05, ** *p* < 0.01, **** *p* < 0.0001.

To further optimize the process, we considered reducing processing steps and reagent consumption. In alkaloid extraction studies, strong acids (e.g., HCl, H_2_SO_4_) are commonly used to increase alkaloid solubility in water and remove interferences with apolar solvents. The aqueous solution is then basified with NaOH or NH_4_OH to pH ∼10.0, rendering the alkaloids less soluble in water and more extractable with medium-high polarity solvents such as hexane, dichloromethane, chloroform, and ethyl acetate.^9,20,21,47^ Then, an alternative method proposed acidifying the aqueous solution with citric acid, a safer candidate to replace strong acids,^21^ while retaining NH_4_OH due to its favorable safety rating.^12^

The choice of extracts from the biphasic system for the concentration of alkaloids was due to the fact that it spared the sample degreasing step (cleanup), and GC-MS data showed that EMeOH and EEtOH from this system were less complex than those obtained from the monophase system. This indicates that hexane and heptane, respectively, were sufficient to remove apolar interferents once they were extracted simultaneously with polar solvents (see Supporting Information Figure S4). When compared to their corresponding fractions, there was no difference between those obtained with hexane (FMHEX) and heptane (FEHEP). However, the subsequent fractions with ethyl acetate (FMEtOAc and FEEtOAc) showed a small but significant difference. The same was observed for the fractions obtained exclusively with ethyl acetate (EMEtOAc and EEEtOAc) (Figure 1C and Table S4). EtOAc showed a high capacity to recover the alkaloids, easily replacing hexane and heptane (see Supporting Information Figures S5 and S6). Other studies have already evaluated the substitution of other solvents such as CH_2_Cl_2_, CHCl_3_ in this concentration step.^12,14,21^

### 3.2 PCA Analysis and Green Chemistry Metrics

Principal component analysis (PCA) was used to explore the chemical profiles of the samples, highlighting the main variations between the different extraction conditions. The samples analyzed included EMeOH, FMHEX, FMEtOAc, EMEtOAc, EEtOH, FEHEP, FEEtOAc and EEEtOAc.

In Figure 3, only two major components were needed to explain more than half of the variation between the data, with PC (42%) and PC2 (23%) with additional components explaining a smaller portion of the variance individually. The PCA revealed significant distinctions among the fractions obtained from different extraction methods. The EMeOH sample, common to both TBM and PMT, was positioned in the upper left quadrant (with a high PC2 value), indicating a unique chemical profile likely associated with the extraction of more polar compounds by the hydromethanolic mixture. The EEtOH sample, shared by GBM and PMG, appeared near EMeOH but with a lower PC2 value, suggesting partial similarity, but showing some differentiation due to the use of ethanol and a higher proportion of water (70:30 v/v).

**Figure 3:**
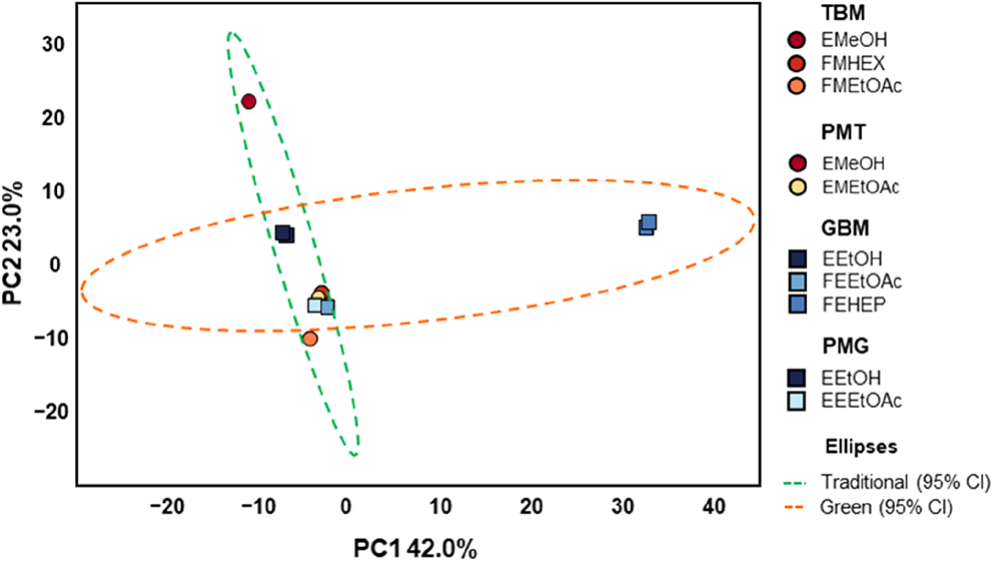
PCA score plot of the chemical profiles of fractions obtained from *W. procera* by traditional (TBM, PMT) and green (GBM, PMG) extraction methods. The ellipses correspond to 95% confidence intervals for samples obtained with traditional and green solvents.

The FMHEX, FMEtOAc, FEEtOAc, EMEtOAc and EEEtOAc fractions clustered closely in the lower left quadrant, indicating high chemical similarity regardless of whether the solvents were traditional or green. This convergence suggests that hexane, heptane, and ethyl acetate exhibit comparable efficiency in extracting apolar and semipolar compounds. In contrast, the FEHEP sample — exclusive to the GBM method — stood out as the most chemically distinct, positioned on the far right of PC1. In the FEHEP fraction, which was partitioned with heptane, the GC-MS analysis showed that aliphatic compounds were still present even after all the fractionation, which was also common in other work with *W. procera*.^6^ This separation highlights the unique profile of metabolites extracted by heptane, underscoring its selective and complementary role in recovering highly apolar compounds. Overall, the results demonstrate that substituting traditional solvents with greener alternatives, such as ethanol and heptane, does not compromise chemical selectivity or diversity, in accordance with the GAC principles.^12^

These compositional distinctions revealed by PCA underscore the importance of assessing not only the chemical coverage of each method but also their environmental and operational sustainability. At the same time, green metrics calculators were used to measure the environmental impact of each method, aligning the experimental design with the principles of green chemistry. The results of these analyses enable a more comprehensive discussion on the relationship between the chemical diversity of extracts and their environmental footprint, thereby guiding the selection of more sustainable methods.

Thus, the use of distinct tools to evaluate the sustainability of analytical methods, such as MoGAPI, AGREE, and AGREEprep, enabled a comprehensive assessment of each method in relation to the 12 GAC Principles. This is because each metric targets different stages of the analytical process. For instance, MoGAPI^39^ primarily emphasizes logistics, safety, and environmental impact, assigning differentiated weights to factors such as solvent toxicity, waste generation, and occupational hazards. AGREE^16^ provides a holistic assessment of the analytical workflow by translating the 12 principles of Green Analytical Chemistry into a visual score, with equal weighting across principles, which allows an overall sustainability overview of the method. In contrast, AGREEprep^40^ is specifically oriented toward sample preparation, giving greater weight to solvent and reagent consumption, energy efficiency, and operational simplicity in the preparation steps. Together, these tools complement each other and provide a transparent and comprehensive sustainability evaluation of the extraction protocols. Figure 4 shows the scores obtained for each method, along with the respective criteria defined by each tool. Detailed descriptions of the parameters evaluated by these tools are provided in the Supporting Information (see Supporting Information Tables S1–S3).

**Figure 4:**
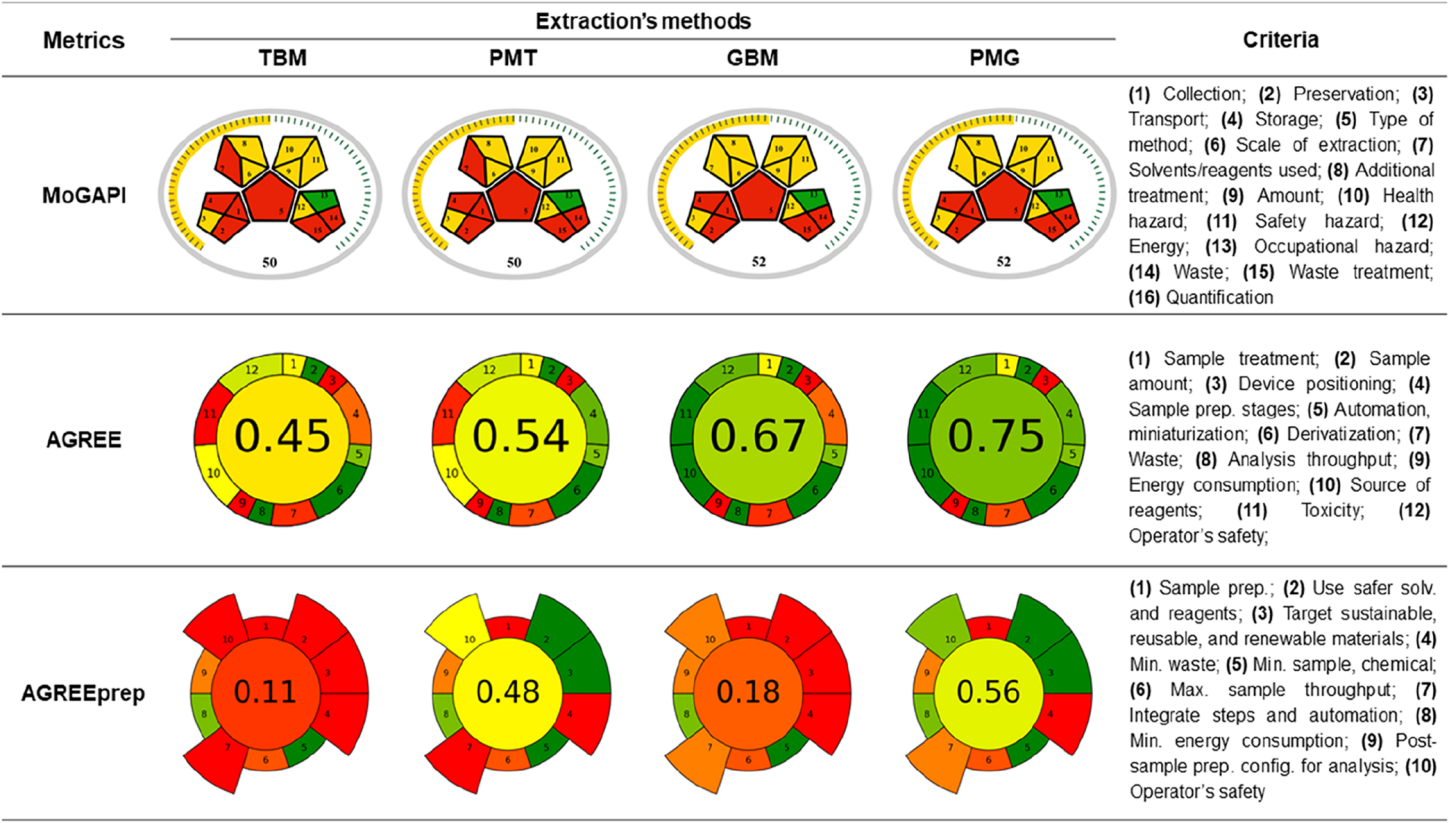
Scores obtained from the application of the different metric tools for traditional (TBM), traditional with modifications (PMT), green (GBM) and green with modifications (PMG) methods.

MoGAPI is a modified version of the GAPI tool, incorporating a final score assignment to facilitate comparison with other assessment tools.^39^ According to this metric, none of the extraction methods fully addressed the risks associated with transport, storage, or occupational hazards. The scores were similar: 50 (TBM and PMT) and 52 (GBM and PMG), with the latter methods benefiting from the substitution of hexane with heptane aligning with Green Analytical Chemistry (GAC) Principle 9.^12^ However, MoGAPI penalized all methods for generating waste >10 mL and required hermetic sealing (NFPA 3 hazards), reflecting its emphasis on logistical and safety risks over analytical efficiency. The replacement of conventional solvents with greener alternatives and the reduction in the number of extraction steps significantly improved the sustainability of the method.

The replacement of the hydromethanolic mixture (80:20 v/v) with a hydroethanolic mixture (70:30 v/v) eliminated methanol, a toxic solvent, and increased water content. Water and ethanol are highly recommended solvents because they are renewable and inexpensive. This substitution improved AGREE scores for GBM (0.67) and PMG (0.75), driven by ethanol’s renewable nature (Principle 7). Similarly, replacing hexane with heptane reduced neurotoxicity risks, as reflected in AGREEprep’s safety scores (criteria 2: PMG: 0.75/1 vs TBM: 0/1). The use of heptane in the extraction processes was due to its similar chemical properties to hexane, safety, and lower toxicity.^48,49^ Hexane is highly volatile, and exposure to it can cause damage to the central nervous system and, in extreme cases, peripheral neuropathy. Additionally, hexane can be more persistent in the environment and contribute to water and air pollution. Heptane, however, tends to degrade more readily, reducing its environmental impact.^50,51^

The most hazardous reagents used were pH modifiers (H_2_SO_4_, NaOH and NH_4_OH), which were used in minimal amounts (0.5 mL each). The pH modifiers were also replaced in the green methods; citric acid and ammonium hydroxide were used as alternatives to sulfuric acid and sodium hydroxide, respectively. The use of acids and bases has a negative impact on the sustainability of the method, so it is recommended to replace or eliminate this step.^52,53^ In the green methods have been replaced with agents that are less harmful to the environment and the analyst. Overall, a critical issue was the need for hermetic sealing, as some methods involve solvents classified as NFPA 3 hazards. The tool does not consider the quantity of these hazardous substances, only their presence in the process. However, in AGREEprep, which specializes in assessing sample preparation, all extraction methods received low scores for waste management (>50 mL) and energy efficiency due to the use of GC-MS.

The PMG method achieved the highest overall score (0.75), driven by the use of green solvents such as EtOH:water (70:30 v/v) and heptane, the elimination of acids, and a more efficient workflow—comprising four steps instead of the seven required in TBM and GBM.^17^ These modifications reduced solvent consumption by 33.5%, minimizing risks and aligning with Principles 3 (safer processes) and 8 (safer solvents). However, the MoGAPI tool does not differentiate between methods in this regard, as all of them generate waste volumes >10 mL. Similarly, AGREEprep penalizes waste generation >50 mL; however, the final score is balanced by the proportion of hazardous, sustainable, renewable, and/or reusable materials used (AGREEprep criteria 2 and 3).

An extraction protocol was optimized without the addition of acids, in compliance with GAC Principles 5 and 1. These principles deal with reducing the use of reagents and avoiding the generation of waste for disposal, respectively.^11,12,55^ In the extraction of alkaloids, these reagents are important because the solubility of these compounds is altered according to the change in pH. Modified methods using traditional and alternative solvents have eliminated the use of any type of acid, since this process is carried out only to remove apolar compounds in the alkaloid concentration stage, thus reducing the number of stages and consequently the volume of waste for disposal.

The PMT and PMG methods utilized only 60.5 mL of solvents (a 66.5% reduction) and eliminated acid usage. Waste reduction was notable in PMT/PMG (60.5 mL vs TBM/GBM: 91 mL), yet all methods failed AGREEprep’s waste criterion (>50 mL = 0). This strategy is in line with GAC Principle 7, which emphasizes the use of renewable raw materials whenever technically and economically possible, and Principle 8, which promotes minimizing or eliminating the use of solvents or reagents classified as hazardous, opting for those that are safer.^11,12,55^ Liquid–liquid extractions are an important alternative for the recovery of organic compounds from complex mixtures.^14,55–57^

Eliminating acids in PMG/PMT simplified workflows (only 3 steps) and aligned with Principle 1 (waste prevention). AGREEprep rewarded this simplification (PMG: 0.25/1 for automation vs TBM: 0/1), while AGREE emphasized its impact on toxicity scores (PMG: 1/1 vs TBM: 0/1). However, MoGAPI’s uniform penalty for waste (>10 mL) prejudiced PMG’s progress, illustrating its limitations in recognizing incremental progress. The PMG method achieved the highest overall AGREE score (0.75) by integrating green solvents, eliminating acids, and reducing steps, yet challenges persist. The Table 1 below cross-references criteria evaluated by AGREE, AGREEprep, and MoGAPI with the 12 GAC Principles, highlighting how each method (TBM, PMT, GBM, PMG) aligns with or violates these guidelines

**Table 1:**
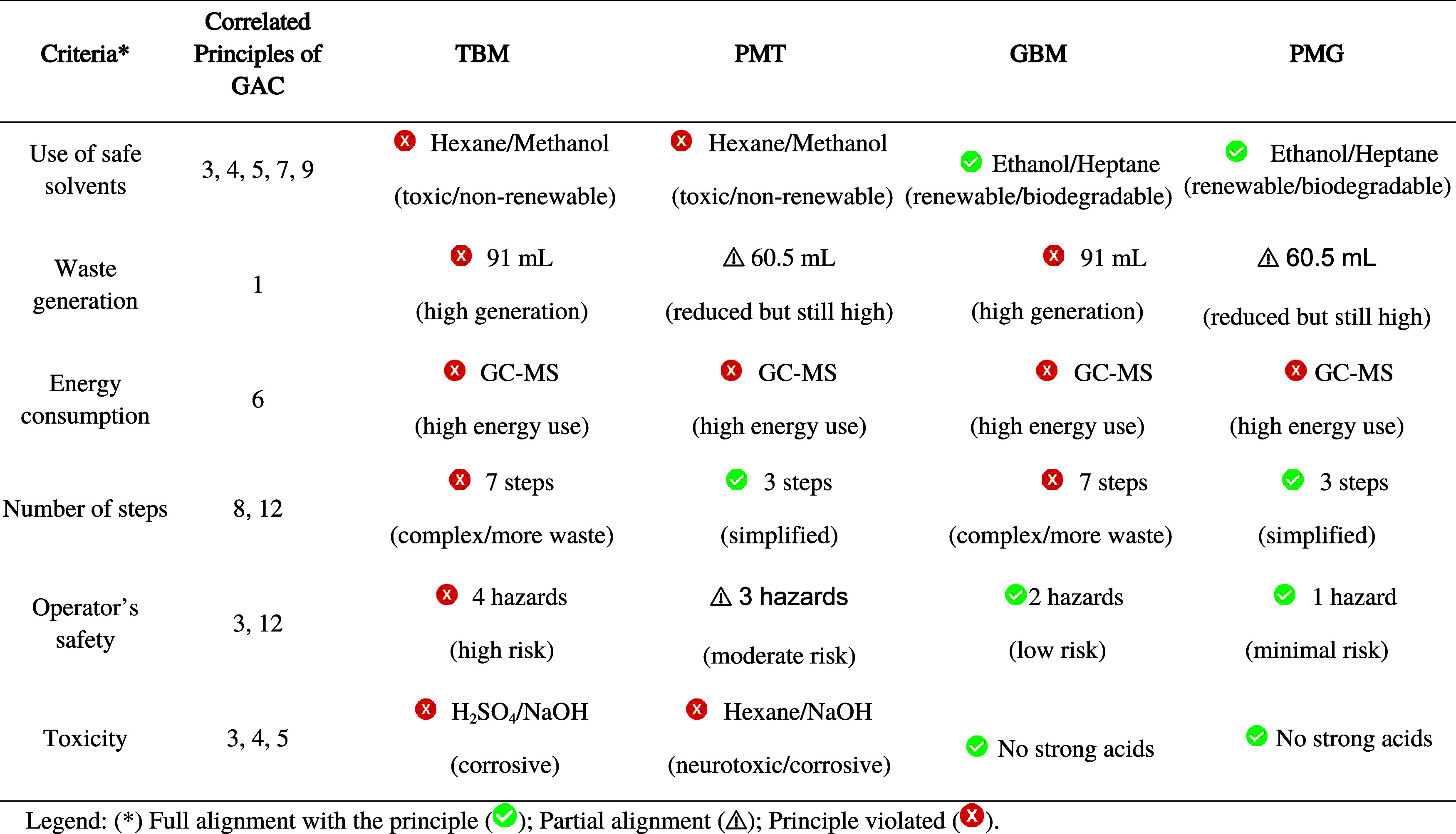
General Criteria Across Metrics Mapped to the 12 Green Analytical Chemistry Principles

This multimetric analysis confirms that PMG represents the most sustainable method, adhering to Principles 3, 5, 7, 8, and 12 through solvent substitutions, hazard reduction, and workflow simplification. However, no method fully satisfied all green chemistry principles, particularly waste prevention and energy efficiency. The integration of AGREE, AGREEprep, and MoGAPI underscores the necessity of holistic assessments to avoid greenwashing and drive innovations such as miniaturized extraction techniques and low-energy analytical instruments. Future work must address these gaps to achieve true sustainability in analytical workflows.

By contextualizing AGREE, AGREEprep and MoGAPI within the 12 principles, this study underlines the value of holistic assessments to avoid greenwashing and drive meaningful progress in sustainable analytical chemistry. By correlating the chemical diversity observed in the extracts by PCA with the calculated green metrics, it was possible to assess the efficiency and environmental impact of each extraction approach. This integration provided a robust framework for selecting methods that not only optimize chemical yields but also adhere to the principles of green chemistry, reducing waste and the use of hazardous solvents.

### 3.3 Chemical Analysis of *Worsleya procera* Leaves

Some samples of *W. procera* exhibited distinct chemical profiles; however, others showed similarity among themselves (see Supporting Information Figures S4 and S5). Compound distribution was visualized using an UpSet plot (Figure 5), where this representation enabled a clear view of the intersections among the chemical profiles of samples obtained through different extraction methods: TBM, GBM, PMT, and PMG. The EMeOH and EEtOH samples, which are shared by the TBM/PMT and GBM/PMG methods, respectively, were considered only once, as they presented overlapping chemical profiles.

**Figure 5:**
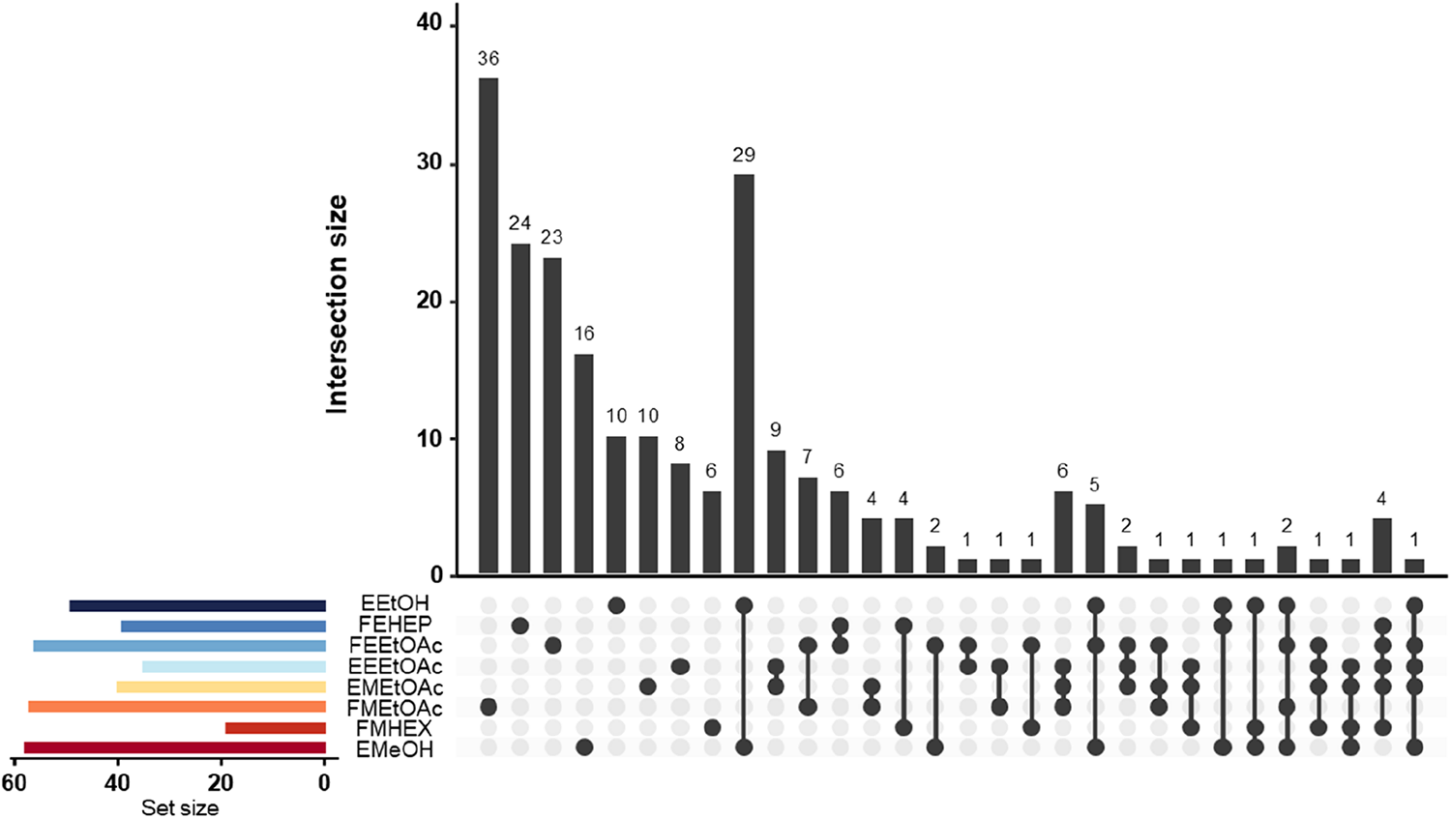
Upset plot of *Worsleya procera* samples obtained by biphasic extraction and fractionation using conventional (EMeOH, FEHEX, FMEtOAc, EMEtOAc) and green (EEtOH, FEHEP, FEEtOAc, EEEtOAc) solvents.

The analysis of intersections among fractions and extracts, as visualized through the UpSet plot, revealed distinct patterns of chemical selectivity and complementarity across the evaluated extraction methods. The FMEtOAc, obtained by the TBM, exhibited 36 unique compounds, highlighting its strong extractive capacity, particularly for semipolar and apolar compounds. Similarly, the FEHEP and FEEtOAc, derived from the GBM, contained 24 and 23 exclusive compounds, respectively, demonstrating that replacing conventional solvents with greener alternatives such as heptane and ethanol did not compromise extraction efficiency. The crude extracts EMeOH and EEtOH, common to TBM/PMT and GBM/PMG respectively, exhibited 16 and 10 unique compounds, indicating broad but less selective chemical profiles. Fractions from PMT and PMG such as EMEtOAc (PMT, 10 unique compounds) and EEEtOAc (PMG, 8 unique) respectively, also showed relevant selectivity despite simplified workflows. FMHEX presented 6 unique compounds, indicating its role in recovering highly apolar substances.

Among the intersections, the most prominent was between EMeOH and EEtOH, with 29 shared compounds, reflecting substantial chemical overlap between hydroalcoholic extracts. Other significant intersections included EEEtOAc and EMEtOAc (9 compounds), FEEtOAc and FMEtOAc (7), FEEtOAc and FEHEP (6), FMEtOAc and EMEtOAc (4), and FEHEP and FMHEX (4), underscoring the complementarity among fractions from different methods. Minor intersections (1–2 compounds) between FEEtOAc and fractions such as EMeOH, EMEtOAc, EEEtOAc, and FMHEX indicate recurrent yet narrower chemical cores. Additionally, multifraction overlaps were observed, such as 6 shared compounds among EEEtOAc, EMEtOAc, and FMEtOAc, and 5 compounds among EEtOH, FEEtOAc, and EMeOH, suggesting repeated chemical profiles across methods.

Overall, biphasic methods (TBM, GBM) provided broader chemical diversity, while modified methods (PMT, PMG) offered greater selectivity and reduced solvent and operational demands. Notably, the PMG method integrates chemical efficiency and sustainability, yielding selective extractions that both concentrate and share relevant compounds also present in conventional approaches. The analysis of *W. procera* extracts revealed that the alkaloid profile varied depending on the extraction method employed, supporting the idea that solvent polarity and fractionation strategy play a decisive role in chemical recovery. A total of 27 alkaloids were detected across different extracts and fractions.

The EMeOH showed a diverse set of galantamine-type and crinine-type alkaloids, including galantamine (**8**), galanthine (**9**), galanthindole (**10**), ismine (**11**), tazettine (**15**), lycorine (**12**). This indicates that MeOH:H_2_O (80:20 v/v) was effective in recovering a broad spectrum of polar and moderately polar alkaloids. FMHEX, being highly apolar, was selective for alkaloids such as albomaculine (**7**), this suggests that apolar solvents can help isolate alkaloids with greater lipophilicity. FMEtOAc, from the methanolic extract, demonstrated the highest alkaloid diversity, with detection of structurally diverse compounds such as 11-deoxytazettine (**2**), 3-O-demetyl-hyppeastidine (**5**), and 5,6-dihydrobicolorine (**6**), in addition to several known bioactive alkaloids like galanthine (**9**), and tazettine (**15**). This highlights the utility of midpolar solvents such as ethyl acetate in concentrating pharmacologically interesting molecules. EMEtOAc concentrated structurally diverse alkaloids including tazettine (**15**), galanthine (**9**), licorine (**12**), galantamine (**8**), and macronine (**13**).

The green extraction method using EtOH:H_2_O (70:30 v/v), EEtOH, resulted in a profile similar to the methanolic extract, recovering galantamine (**8**), galanthine (**9**), ismine (**11**), lycorine (**12**), and tazettine (**15**). Despite being greener, ethanol appeared slightly less efficient in extracting the full diversity of compounds unless followed by partitioning. FEHEP selectively retained lipophilic alkaloids such as albomaculine (**7**). FEEtOAc and EEEtOAc proved especially rich in bioactive alkaloids, including galantamine (**8**), galanthine (**9**), tazettine (**15**), and ismine (**11**) (Table 2 and see Supporting Information Table S5).

**Table 2:** Alkaloids Annotated in *Worsleya procera* Leaves by GC-MS

ID	alkaloids	LRI^a^	LRI^b^	M^+^	ion fragments	refs
1	11,12-dehydrolycorene	2349.8	2360	281 (44)	252 (16), 254 (15), 224 (13), 139 (12), 167(9), 166 (8), 222 (8), 111(8), 140 (7)	^58^
2	11-deoxytazettine	2504.8	2485	316 (2)	231 (100), 230 (27), 44 (20), 232 (16), 315 (13), 300 (13), 197 (13), 211 (13), 42 (13), 227 (12)	^59,60^
3	3-epimacronine	2804.6	2810	329 (14)	245(100), 201 (79), 44 (44), 42 (30), 244 (25), 70 (21), 314 (18), 197(16), 255 (16), 246 (14)	^58,61,62^ N
4	3-*O*-acetylpowelline	2792.8	2768.7	344 (26)	343 (100), 286 (79), 271 (63), 300 (35), 107 (27), 256 (25), 227 (25), 226 (21), 272 (20), 228 (18)	^63^
5	3-*O*-demetyl-hyppeastidine	2673.4		389 (4)	305 (100), 70 (61), 75 (22), 306 (22), 304 (18), 42 (17), 214 (17), 240 (14), 181 (14), 318 (14)	^64^
6	5,6-dihydrobicolorine	2328.4	2321	239(43)	238 (100), 180(16), 139(8), 118(8), 152(7), 90(7), 240(4), 181 (4)	^65^
7	albomaculine	2797.5	2815	345 (<1)	109 (100), 108 (18), 110 (8), 42 (3), 82 (3), 94 (3), 81 (2), 93 (1), 67 (1), 41 (1)	^6,66,67^
8	galantamine	2391	2405	289 (2)	286 (100), 287 (80), 216 (38), 174 (34), 244 (31), 42 (20), 115 (19), 288 (14), 230 (14), 270 (13)	^65,68–70^ N
9	galanthine	2690.2	2704	318 (3)	243 (100), 242 (93), 317 (17), 244 (17), 268 (15), 316 (13), 284 (12), 125 (12), 162 (10), 266 (10)	^59,67,71^ N
10	galanthindole	2492	2487	281 (100)	263 (24), 262 (22), 191 (18), 282 (18), 94 (18), 107 (17), 252 (16), 264 (16) 132 (15)	^6,58,68–70,72^ N
11	ismine	2266	2280	257 (32)	238 (100), 239 (17), 180 (11), 196 (11), 168 (11), 139 (9), 167 (8), 107 (8), 77 (7)	^6,59,66,67^ N
12	lycorine	2748.2	2746	287 (23)	226 (100), 227 (78), 287 (23), 268 (21), 286 (16), 250 (13), 147 (12), 228 (11), 111 (8), 119 (8), 211 (5)	^6,62,66,67,71^ N
13	macronine	2658.6	2689	355 (<1)	70 (100), 245 (30), 42 (26), 314 (17), 201 (16), 44 (15), 301 (9), 329 (8), 203 (7), 286 (6)	^61,71^
14	tazettamide	2900.7	2866	341 (<1)	260 (100), 201 (74), 229 (45), 115 (40), 42 (35), 313 (25), 254 (23), 171 (22), 173 (19), 261 (19)	^74^ N
15	tazettine	2642.4	2651	331 (<1)	247 (100), 70 (40), 44 (35), 42 (33), 71 (32), 45 (30), 115 (21), 298 (20), 331 (17), 201 (17)	^58,59,71^ N
others alkaloid subtypes detected^c^						
16	unidentified type 1	2808.9		341 (<1)	296 (100), 297 (68), 280 (18), 281 (14), 298 (10), 148 (8), 282 (8), 252 (8), 180 (6), 237 (5)	^74^
17	homolycorine type	2879.2		296 (2)	139 (100), 125 (89), 124 (73), 96 (55), 278 (24), 279 (18), 140 (13), 42 (12), 94 (10), 126 (8)	^74^
18	A1	2741.3		282 (<1)	109 (100), 108 (21), 110 (8), 42 (4), 82 (4), 94 (3), 81 (3), 93 (1), 41 (1), 107 (1)	^75^
19	lycorine type	2741.9		288 (1)	109 (100), 226 (45), 227 (34), 108 (23), 110 (9), 268 (9), 287 (8), 82 (8), 42 (8), 250 (6)	^71^
20	nerinine (I) type	2744.7		341 (<1)	109 (100), 226 (90), 227 (68), 108 (23), 287 (19), 268 (18), 286 (12), 250 (12), 147 (11), 82 (11)	^72,73^
21	nerinine (II) type	2798.9		344 (<1)	109 (100), 108 (25), 110 (7), 42 (5), 82 (4), 94 (3), 81 (3), 93 (2), 41 (1), 65 (1)	^72^
22	tazettine (I) type	2850.7		313 (30)	260 (100), 242 (71), 201 (66), 299 (50), 57 (41), 227 (40), 71 (35), 255 (33), 115 (30)	^59^
23	tazettine (II) type	2903.0		355 (<1)	242 (100), 299 (64), 227 (44), 255 (34), 107 (19), 256 (17), 243 (16), 300 (15), 228 (13), 199 (11)	^59^
24	A13	2930.3		295 (90)	294 (100), 296 (17), 147 (13), 278 (13), 250 (11), 279 (8), 280 (8), 178 (7), 221 (6)	^71^
25	A4	3111.6		355 (2)	257 (100), 125 (83), 282 (71), 273 (69), 331 (54), 242 (38), 110 (36), 207 (28), 258 (26), 123 (24)	^75–77^
26	GG-1	2450.9		299 (7)	240 (100), 238 (95), 210 (42), 270 (33), 239 (21), 225 (21), 224 (21), 241 (16), 182 (16), 180 (15)	^69^
27	M+ 269	2219.8		269 (38)	238 (100), 241 (20), 180 (20), 239 (19), 139 (13), 90 (12), 152 (11), 225 (11), 210 (10)	^61^

a LRI: Linear Retention Index experimental.

b LRI teoric; (−) Unknow.

c Alkaloids not yet identified: the name given to the subtype based on the fragmentation pattern characteristic of these alkaloids; N: NIST11 library with SI > 86%.

This highlights that the combined use of safer pH modulators (e.g., citric acid, NH4OH) and green solvents can be an effective alternative to conventional partitioning that typically uses chloroform or dichloromethane and others.^9,20,21,47^ Overall, the green extraction strategy not only allowed selective recovery of key bioactive compounds, but also provided a platform for rational alkaloid partitioning without compromising analytical or pharmacological relevance. The approach demonstrates the feasibility of sustainable extraction protocols for Amaryllidaceae alkaloids, with great potential for optimization based on compound polarity, solvent systems, and downstream applications. These substances have been identified in other species of the Amaryllidaceae family and are under constant investigation, with biological activities such as anti-inflammatory, analgesic, antimicrobial, antiparasitic, anticonvulsant, antidepressant, antiviral, antitumor and anticholinesterase attributed through various studies.^4,7^

A study by Gonring-Salarini et al.^6^ has been published, chemically characterizing the first part of this species, the authors reported the annotation of 18 alkaloids present in the roots of this species, where compounds isolated from the roots showed antiplasmodial and antitumor activity in vitro. This is the first known published study of the substances present in the aerial parts, making it highly relevant to the chemical knowledge of *W. procera* (see Supporting Information, Table S4).

Eight annotated alkaloids coincided with those found in the roots of *W. procera*.^6^ The FMEtOAc, FEEtOAc and EEEtOAc samples had the highest number of alkaloids identified, including: albomaculine (**7**), lycorine (**12**), 3-epimacronine (**3**), tazettine (**15**), galantine and galantamine (**8**), the latter two being the only substances present in all samples (Figure 6).

**Figure 6:**
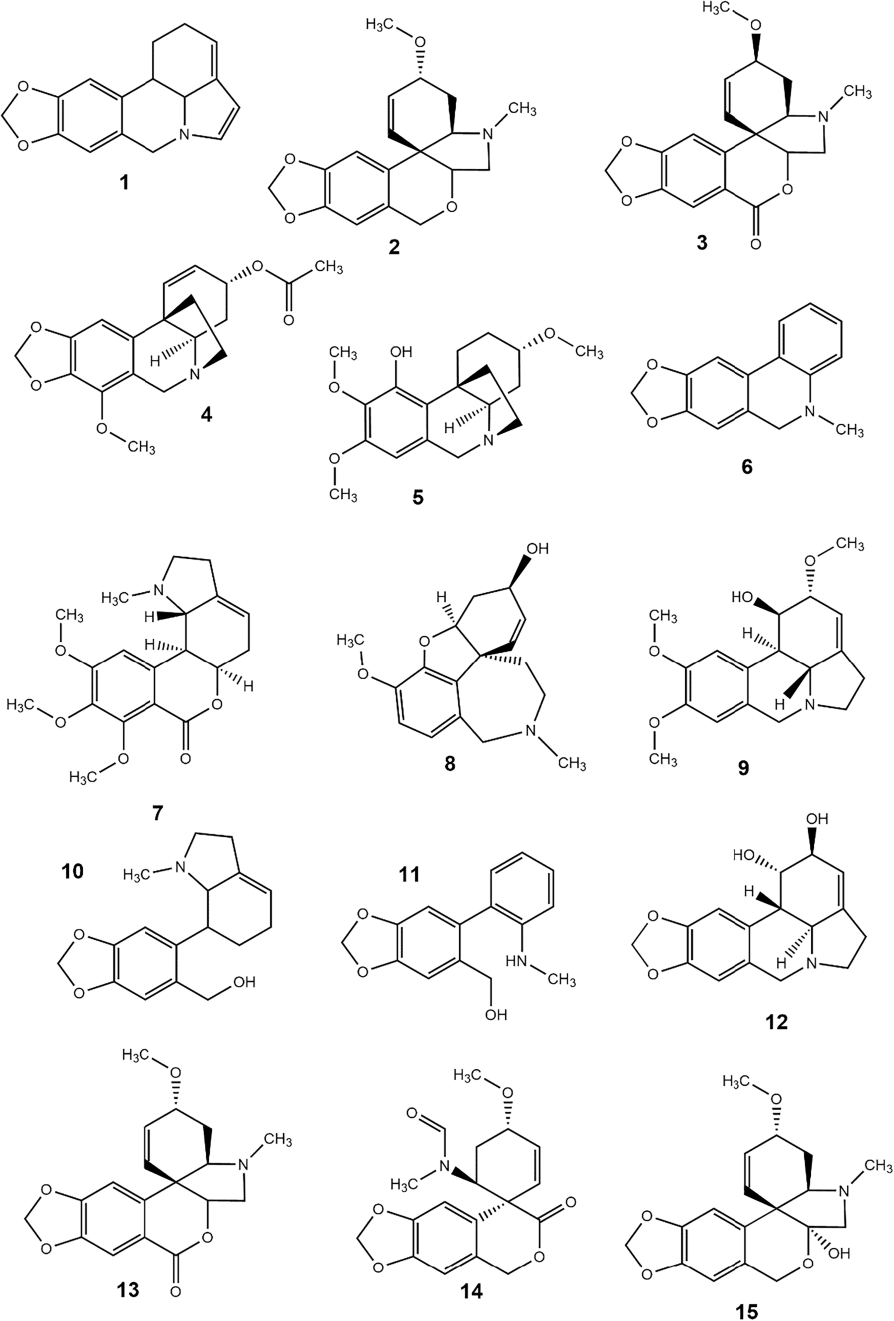
Alkaloid chemical structures annotated by GC-MS analysis. (**1**) 11,12-dehydrolycorene, (**2**) 11-deoxytazettine, (**3**) 3-epimacronine, (**4**) 3-O-acetylpowelline, (**5**) 3-O-demetyl-hippeastidine, (**6**) 5,6-dihydrobicolorine, (**7**) albomaculine, (**8**) galantamine, (**9**) galanthine, (**10**) galanthindole, (**11**) ismine, (**12**) lycorine, (**13**) macronine, (**14**) tazettamide and (**15**) tazettine.

It is worth noting that, although GC-MS is widely used and many studies report only this type of analysis, the technique has intrinsic limitations. Since alkaloids may include nonvolatile constituents, some compounds could remain undetected under GC-MS conditions. Recognizing this limitation, future investigations should incorporate complementary approaches such as LC-MS (low energy consumption, therefore a greener technique) or NMR to provide a more complete characterization of the alkaloid composition.

### 3.4 Anticholinesterase Activity

For this purpose, the bioaffinity chromatography technique was used, where the samples were coinjected with acetylcholine and butyrylcholine in an HPLC-MS system with the immobilized enzymes: electric fish acetylcholinesterase (_eel_AChE-ICER) and human butyrylcholinesterase (_hu_BChE-ICER). The inhibition percentage is determined by comparing the enzyme activity area with and without the inhibitor, subtracting the choline area from the substrate’s spontaneous hydrolysis, controlled using an empty capillary.^36–38^ The results are presented in Figure 7.

**Figure 7:**
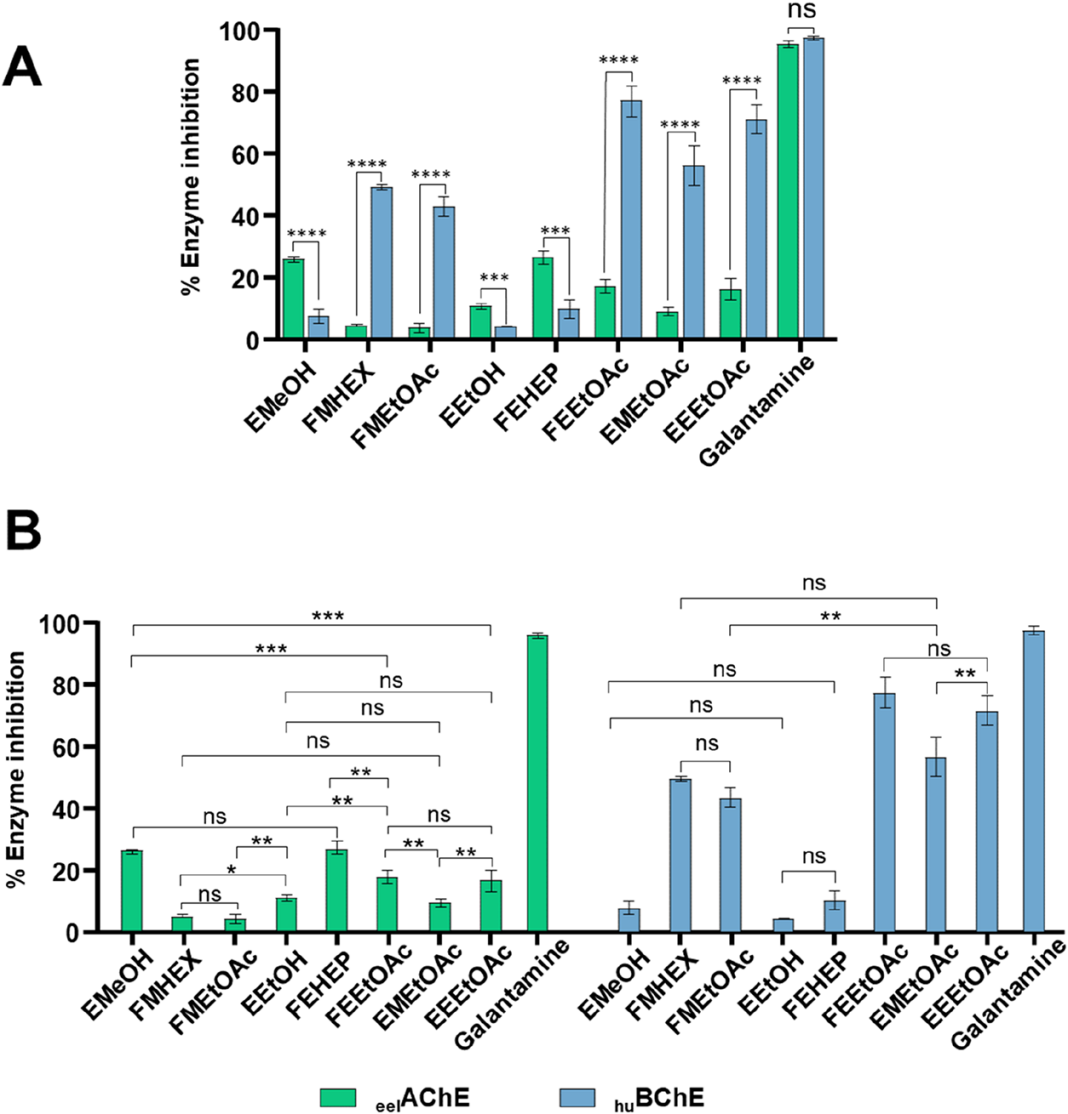
Inhibitory activity of *Worsleya procera* extracts and fractions against acetylcholinesterase (_eel_AChE) and butyrylcholinesterase (_hu_BChE). Legend: (A) Overall comparison of enzyme inhibition (%) between _eel_AChE and _hu_BChE for each tested sample. (B) Comparative analysis among the samples, separated into two groups according to the enzyme evaluated: on the left, comparison of samples against _eel_AChE; on the right, comparison of samples against _hu_BuChE. In this graph, additional comparisons not displayed showed highly significant differences (****, *p* < 0.0001). Values are expressed as mean ± SD (*n* = 3). Statistical differences are indicated as follows: *p < 0.05; **p < 0.01; ***p < 0.001; ****p < 0.0001; ns = not significant.

The evaluation of the anticholinesterase activity of *W. procera* extracts and fractions revealed distinct inhibition profiles of _eel_AChE and _hu_BChE. The inhibitory potential was correlated with the chemical composition previously established by GC-MS and with pharmacological data of isolated compounds reported in the literature. This is particularly relevant since the lack of standardized procedures for obtaining alkaloid-enriched fractions hampers direct comparisons. The positive control, galanthamine (8), inhibited 95.3 ± 1.1% of _eel_AChE and 97.1 ± 0.1% of _hu_BChE, values consistent with previously reported.^36–38^

The hydromethanolic (EMeOH) and hydroethanolic (EEtOH) extracts showed higher anti-ChE activity against AChE, with values of 25.7 ± 0.8% and 10.5 ± 1.0%, respectively. These extracts contain galanthine (9), an alkaloid widely described as a moderate AChE inhibitor,^1,5^ with IC_50_ values between 6.1–7.75 μM,^79^ and BChE with IC_50_ > 60 μM.^80^

The FMHEX and FMEtOAc fractions displayed low anti-AChE activity, corresponding to 4.4 ± 0.4% and 3.6 ± 1.5%, respectively. GC-MS analysis identified mainly albomaculine (7) and, to a lesser extent, galanthine (9). Albomaculine has been reported as inactive against AChE.^78^ However, the inhibitory potential against BChE was significant, with FMHEX inhibiting 49.0 ± 0.8% and FMEtOAc 42.9 ± 3.1%. Although no studies have yet reported anti-BChE activity for albomaculine (7), these findings suggest a possible contribution of this alkaloid.

The FEHEP fraction exhibited anti-AChE activity of 26.3 ± 2.2% and anti-BChE activity of 9.6 ± 3.1%. Despite the presence of albomaculine (7), unidentified compounds likely contributed to the low anti-BChE activity, as this fraction displayed a distinct profile compared with the others (see Supporting Information, Figure S5). Among the identified constituents, 3-epimacronine (3) showed moderate anti-ChE activity, with a preference for AChE (IC_50_ = 89 ± 3 μM), while being considered inactive for BChE (IC_50_ = 425 ± 14 μM).^81^

The EMAcOEt, FEEtOAc, and EEAcOEt fractions exhibited inhibitory potential approximately 17-fold higher for BChE (56.0 ± 6.3%, 76.9 ± 5.0%, and 71.0 ± 4.6%, respectively) compared to AChE (8.8 ± 1.3%, 17.1 ± 2.2%, and 16.0 ± 3.5%, respectively). These fractions contained mainly tazettine (15), galanthine (9), and albomaculine (7). Although tazettine has been previously described as a weak inhibitor (IC_50_ > 200 μM),^80,82^ a more recent study reported an IC_50_ of 4.33 ± 1.28 μM for BChE.^83^ Galanthine (9) also showed activity against BChE, with an IC_50_ of 12.02 μM,^79^ supporting the inhibitory activity observed. Other identified alkaloids, such as lycorine, exhibited only weak inhibition (IC_50_ = 155 μM for AChE;^78^ > 200 μM in other studies,^82^) consistent with the absence of significant effects in the corresponding fractions.

In summary, the integrated analysis demonstrates that AChE inhibition is mainly attributed to galanthine and galanthamine,^53,54^ while BChE inhibition results from the synergistic action of galanthamine and tazettine, in addition to the possible contribution of albomaculine. These findings highlight the need to reassess alkaloids previously considered inactive, such as albomaculine and tazettine, under new experimental conditions, underscoring their potential relevance in advanced stages of Alzheimer’s disease, in which BChE assumes greater pathophysiological importance.

## 4 Conclusions

The present study provided valuable insights into the sustainable extraction of bioactive alkaloids from *W. procera* and their anticholinesterase activity under the principles of Green Analytical Chemistry. In this first systematic study of alkaloids from *W. procera* leaves, 27 alkaloids were detected and 15 annotated, mainly from the licorine, homolicorine, and tazettine subgroups. Among the extraction methods evaluated, the optimized biphasic method without acid addition (PMG) proved to be both chemically effective and the most sustainable, reducing solvent use while generating fractions with strong _hu_BChE inhibitory potential (>70%). These findings reinforce the importance of adopting green methodologies in natural product research, demonstrating that sustainability can be achieved without compromising biological activity. Moreover, the developed approach provides a promising foundation for broader applications, from the discovery of novel therapeutic agents for neurodegenerative diseases to strategies supporting the sustainable use and conservation of biodiversity.

## Supplementary Material



## Notes

Proof of registration in the Sistema Nacional de Gestão de Patrimônio Genético e do Conhecimento Tradicional Associado; schematic representations of monophasic, biphasic, and modified extraction systems; chromatographic profiles of apolar and polar extracts and fractions obtained by different extraction approaches; results of MoGAPI, AGREE, and AGREEprep evaluations for traditional, modified, and green methods; yields of *Worsleya procera* leaf extracts and alkaloid-enriched fractions; annotation of alkaloids by GC-MS with confidence levels; and relative intensities of compounds in extracts and fractions by GC-MS (PDF)

## Abbreviations

AChE: acetylcholinesterase
_eel_AChE: enzymes electric fish acetylcholinesterase
AGREE: Analytical GREEnness Evaluation
AGREEprep: Analytical Greenness Metric for Sample Preparation
Anti-ChE: anticholinesterase
BChE: butyrylcholinesterase
_hu_BChE: enzymes human butyrylcholinesterase
EtOH: ethanol
EtOAc: ethyl acetate
GAC: Green Analytical Chemistry
GAPI: Green Analytical Procedure Index
H_2_O: water
HCl: chlorhydric acid
H_2_SO_4_: sulfuric acid
ICER: Immobilized Capillary Enzyme Reactor
LRI: Linear Retention Index
MoGAPI: Modified Green Analytical Produce Index tool
GBM: green biphasic method
GMM: Green Monophasic Method
MeOH: methanol
NaOH: sodium hydroxide
NH_4_OH: ammonium hydroxide
PMG: partitional modified green
PMT: partitional modified green
TBM: traditional biphasic method
TMM: traditional monophasic method
EMeOH: methanolic extract
FMHEX: fraction obtained with hexane from the hydromethanolic extract
FMEtOAc: fraction obtained with ethyl acetate from the hydromethanolic extract
EEtOH: ethanolic extract
FEHEP: fraction obtained with heptane from the hydroethanolic extract
FEEtOAc: fraction obtained with ethyl acetate from the hydroethanolic extract
EMEtOAc: fraction obtained with ethyl acetate by the modified method from the hydromethanolic extract
EEEtOAc: fraction obtained with ethyl acetate by the modified method from the hydroethanolic extract
PCA: Principal Component Analysis
