# Supplementary material for: Sustainable Extraction of Alkaloids from Worsleya procera: Improving the Method with Green Chemistry
Source: ACS Omega. 2025 Nov 4;10(45):54834–49. doi: 10.1021/acsomega.5c08468 (PMC12631677; doi:10.1021/acsomega.5c08468)
Supplement: Supplementary file 1 [file ao5c08468_si_001.pdf]

Supporting Information for

Sustainable extraction of alkaloids from *Worsleya procera*: Improving the method with Green Chemistry

Winner Duque Rodrigues[†*]; Ana Caroline Zanatta[†]; Tiago Cabral Borelli[‡]; Ricardo Roberto da Silva[‡]; Carmen Lucia Cardoso[§]; Norberto Peporine Lopes[†*]

[†] Research Center for Natural and Synthetic Products - School of Pharmaceutical Sciences of Ribeirão Preto, University of São Paulo, Ribeirão Preto - SP, 14040-900, Brazil.

[‡] Computational Chemical Biology Laboratory - School of Pharmaceutical Sciences of Ribeirão Preto, University of São Paulo, Ribeirão Preto - SP, 14040-900, Brazil.

[§] Bioaffinity Chromatography and Natural Products Group, School of Philosophy, Sciences, and Letters of Ribeirão Preto, University of São Paulo, Ribeirão Preto, 14040-90, Brazil.

*Corresponding authors: winnerduque@usp.br; npelopes@fcfrp.usp.br

Contents (13 pages, 1 registration declaration, 6 figures and 6 tables)

DOC S1: Proof of registration in the Sistema Nacional de Gestão de Patrimônio Genético e do Conhecimento Tradicional Associado…………………………S3

Figure S1: Schematic representation of methods using traditional and alternative solvents in (A) monophasic and (B) biphasic extraction systems………………S4

Figure S2: Schematic representation of methods using traditional and alternative solvents in modified extraction system……………………………………………..S5

Figure S3: Chromatographic profile of the apolar extracts obtained through monophasic and biphasic extraction with hexane (A and B) and heptane (C and D), respectively……………………………………………………………………S5

Figure S4: Chromatographic profile of the polar extracts obtained through monophasic and biphasic extraction with hydromethanol (80:20 v/v) (A and B) and hydroethanol (70:30 v/v) (C and D), respectively……………………………S6

Figure S5: Chromatographic profile of fractions from the hydromethanolic extract: partitioning with hexane (FMHEX), ethyl acetate (FMEtOAc), and direct fraction with ethyl acetate (EMEtOAc); fractions from the hydroethanolic extract: partitioning with heptane (FEHEP), ethyl acetate (FEEtOAc), and direct fraction with ethyl acetate (EEEtOAc)……………………………………………………S6

Figure S6: Chromatographic profiles of the polar extracts obtained after alkaloid extraction using monophasic and biphasic systems with hydromethanol (80:20 v/v) (A and B) and hydroethanol (70:30 v/v) (C and D), respectively……………S7

Table S1: Results obtained from the application of the MoGAPI tool for traditional (TBM), traditional with modifications (PMT), green (ABM) and green with modifications (PMG) methods……………………………………………………S8

Table S2: Results obtained from the application of the AGREE tool for traditional (TBM), traditional with modifications (PMT), green (GBM) and green with modifications (PMG) methods………………………………………………….....S9

Table S3: Results obtained from the application of the AGREEprep tool for traditional (TBM), traditional with modifications (PMT), green (GBM) and green with modifications (PMG) methods……………………………………………..S10

Table S4: Yield of *Worsleya procera* leaf extracts and alkaloid-enriched fractions…………………………………………………..…………………..S11

Table S5: Annotation of alkaloids in *Worsleya procera* leaves by GC-MS and their confidence levels……………………………………………..……………….S11

Table S6: Relative intensities of compounds in extracts and different fractions of *Worsleya procera* leaves by GC-MS…………………….…………………….S12

Ministério do Meio Ambiente
CONSELHO DE GESTÃO DO PATRIMÔNIO GENÉTICO
SISTEMA NACIONAL DE GESTÃO DO PATRIMÔNIO GENÉTICO E DO CONHECIMENTO TRADICIONAL ASSOCIADO

Certidão
Cadastro nº AE355E3

Declaramos, nos termos do art. 41 do Decreto nº 8.772/2016, que o cadastro de acesso ao patrimônio genético ou conhecimento tradicional associado, abaixo identificado e resumido, no Sistema Nacional de Gestão do Patrimônio Genético e do Conhecimento Tradicional Associado foi submetido ao procedimento administrativo de verificação e não foi objeto de requerimentos admitidos de verificação de indícios de irregularidades ou, caso tenha sido, o requerimento de verificação não foi acatado pelo CGen.

Número do cadastro:	**AE355E3**
Usuário:	**Winner Duque Rodrigues**
CPF/CNPJ:	**013.827.526-28**
Objeto do Acesso:	**Patrimônio Genético**
Finalidade do Acesso:	**Pesquisa**

Espécie

Worsleya procera
Griffinia sp
Cearanthes sp

Título da Atividade:	**Análise metabolômica da quimiodiversidade presente na família Amaryllidaceae e prospecção da atividade anticolinesterásica**

Equipe

**Winner Duque Rodrigues**	**Universidade de São Paulo**
**Norberto Peporine Lopes**	**Universidade de São Paulo**
**Carmen Lucia Cardoso**	**Univerdidade de São Paulo**

Data do Cadastro:	**31/05/2023 22:15:36**
Situação do Cadastro:	**Concluído**

Conselho de Gestão do Patrimônio Genético
Situação cadastral conforme consulta ao SisGen em **20:22** de **26/06/2025**.

SISTEMA NACIONAL DE GESTÃO
DO PATRIMÔNIO GENÉTICO
E DO CONHECIMENTO TRADICIONAL
ASSOCIADO - SISGEN

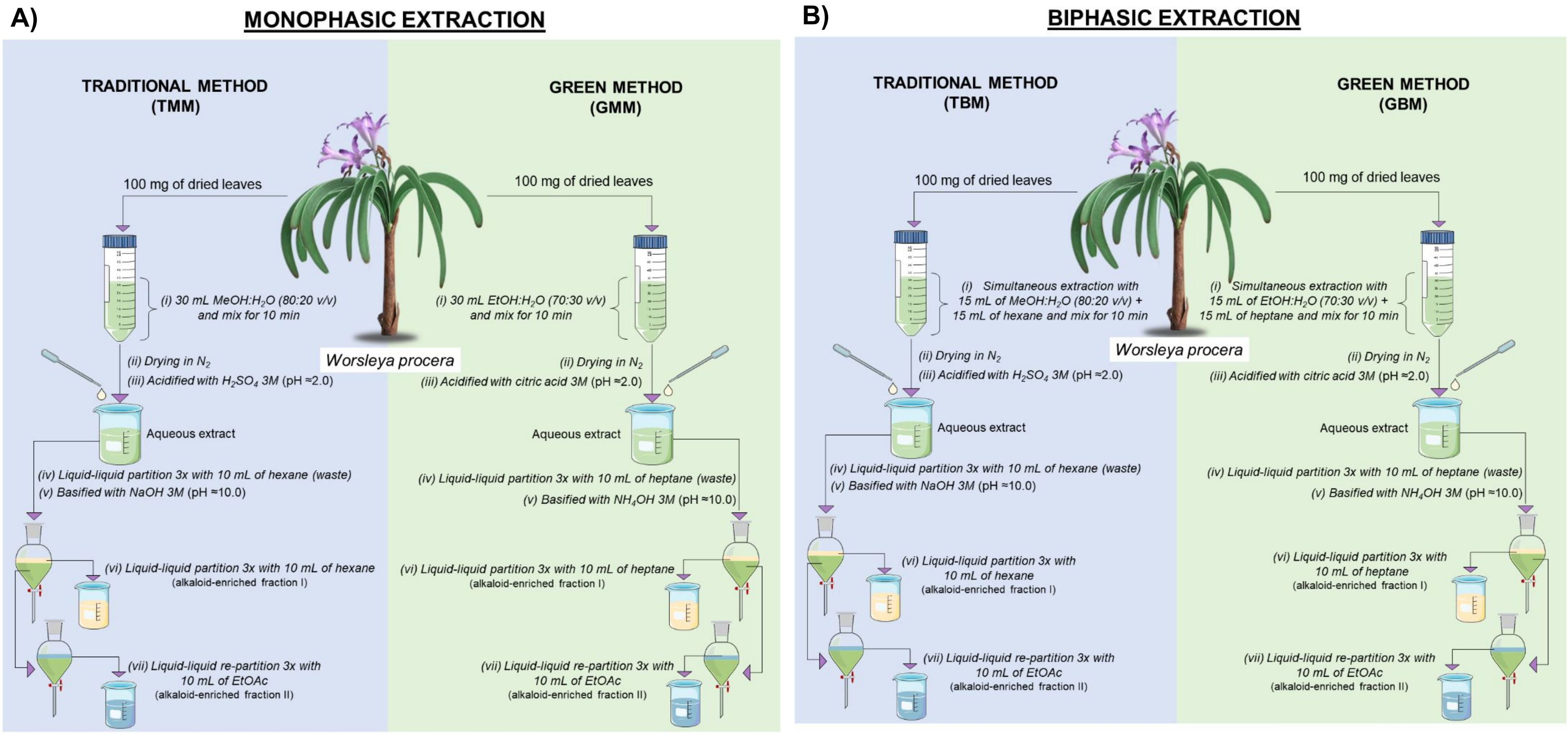

Figure S1: Schematic representation of methods using traditional and alternative solvents in (A) monophasic and (B) biphasic extraction systems.

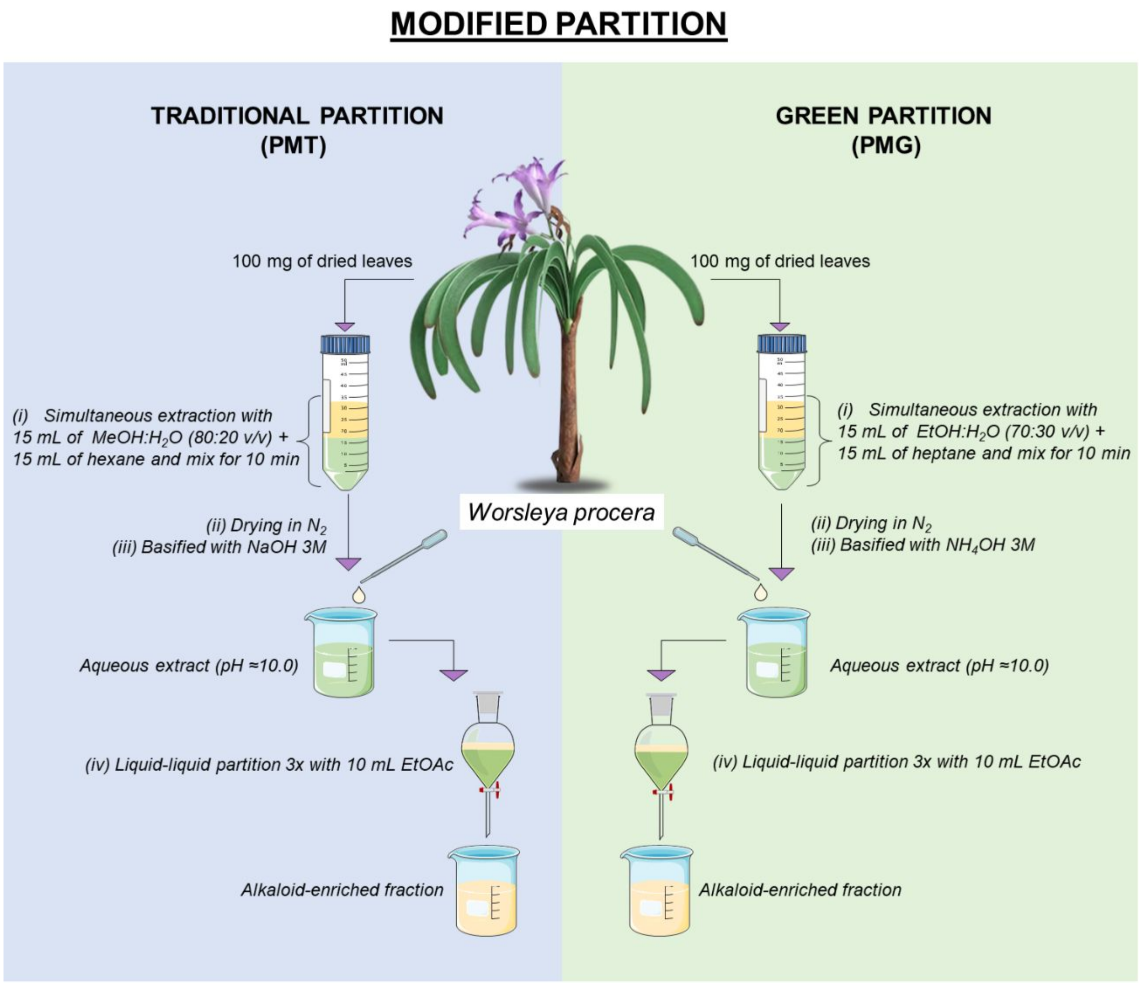

Figure S2: Schematic representation of methods using traditional and alternative solvents in modified extraction system.

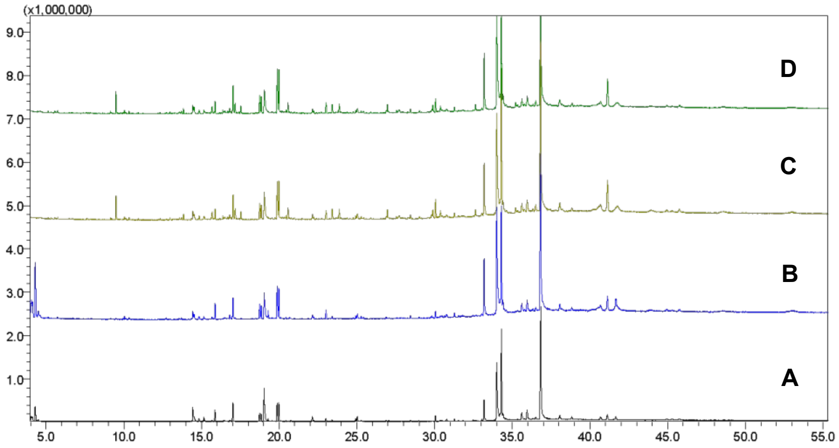

Figure S3: Chromatographic profile of the apolar extracts obtained through monophasic and biphasic extraction with hexane (A and B) and heptane (C and D), respectively.

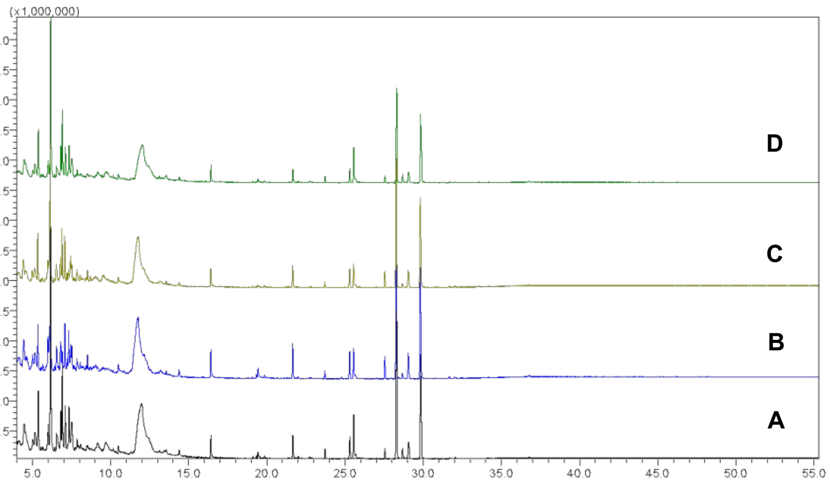

Figure S4: Chromatographic profile of the polar extracts obtained through monophasic and biphasic extraction with hydromethanol (80:20 v/v) (A and B) and hydroethanol (70:30 v/v) (C and D), respectively.

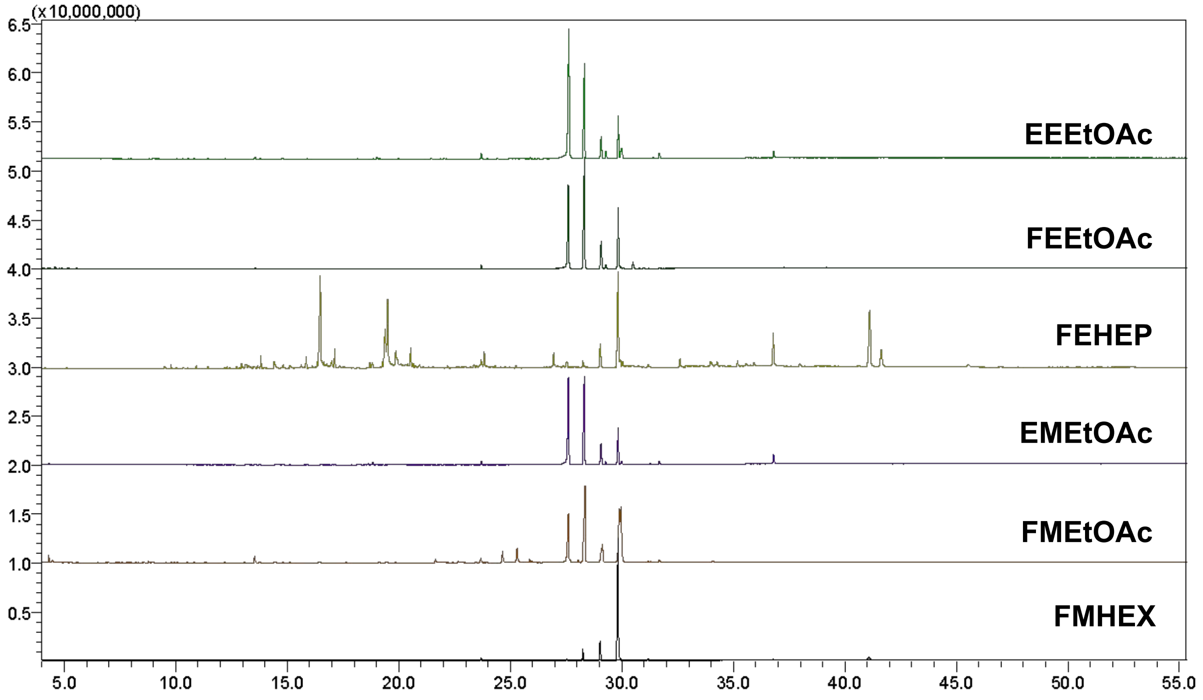

Figure S5: Chromatographic profile of fractions from the hydromethanolic extract: partitioning with hexane (FMHEX), ethyl acetate (FMEtOAc), and direct fraction with ethyl acetate (EMEtOAc); fractions from the hydroethanolic extract: partitioning with heptane (FEHEP), ethyl acetate (FEEtOAc), and direct fraction with ethyl acetate (EEEtOAc).

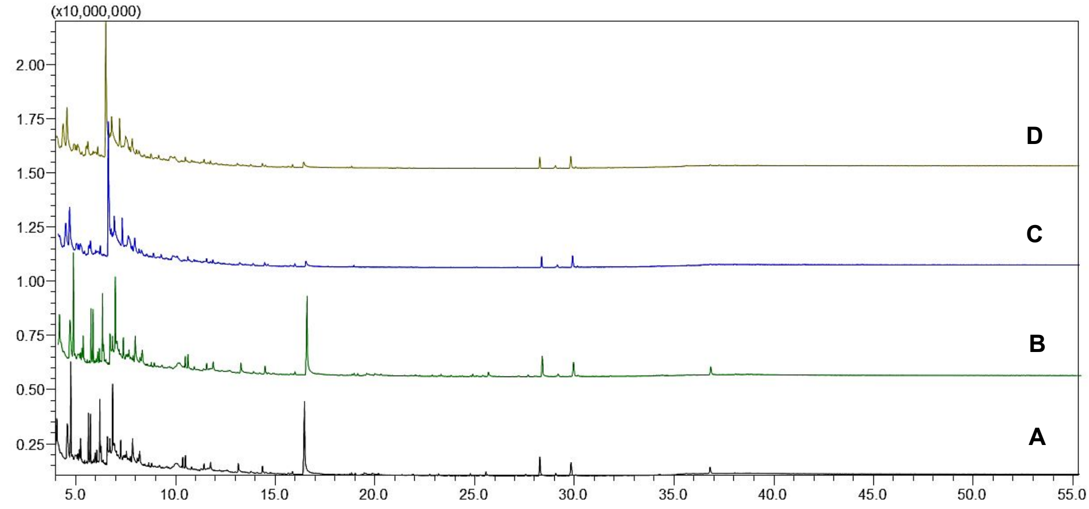

Figure S6: Chromatographic profiles of the polar extracts obtained after alkaloid extraction using monophasic and biphasic systems with hydromethanol (80:20 v/v) (A and B) and hydroethanol (70:30 v/v) (C and D), respectively.

Table S1: Results obtained from the application of the MoGAPI tool for traditional (TBM), traditional with modifications (PMT), green (ABM) and green with modifications (PMG) methods.

Criteria	Extraction's methods			
	**TBM**	**PMT**	**GBM**	**PMG**
	**Data**	**Data**	**Data**	**Data**
1. Collection	Off-line	Off-line	Off-line	Off-line
2. Preservation	Physico-chemical	Physico-chemical	Physico-chemical	Physico-chemical
3. Transport	Required	Required	Required	Required
4. Storage	Under special conditions	Under special conditions	Under special conditions	Under special conditions
5. Type of method	Extraction required	Extraction required	Extraction required	Extraction required
6. Scale of extraction	Micro-extraction	Micro-extraction	Micro-extraction	Micro-extraction
7. Solvents/reagents used	Non-green solvents/reagents	Non-green solvents/reagents	Green solvents/reagents	Green solvents/reagents
8. Additional treatment	Simple treatments	Simple treatments	Simple treatments	Simple treatments
9. Amount	10-100 mL (or g)	10-100 mL (or g)	10-100 mL (or g)	10-100 mL (or g)
10. Health hazard*	NFPA=3; sulfuric acid (H314) and sodium hydroxide (H314)	NFPA=3; sodium hydroxide (H314)	NFPA=3; ammonium hydroxide (H314, H400)	NFPA=3; ammonium hydroxide (H314, H400)
11. Safety hazard*	NFPA=3; Dominant hazardous agents: H_2SO_4 and NaOH	NFPA=3; Dominant hazardous agent: NH_4OH	NFPA=3; Dominant hazardous agent: NaOH	NFPA=3; Dominant hazardous agent: NH_4OH
12. Energy	≤ 1.5 kWh	≤ 1.5 kWh	≤ 1.5 kWh	≤ 1.5 kWh
13. Occupational hazard	Hermetic sealing of analytical process	Hermetic sealing of analytical process	Hermetic sealing of analytical process	Hermetic sealing of analytical process
14. Waste	> 10 mL	> 10 mL	> 10 mL	> 10 mL
15. Waste treatment	No treatment	No treatment	No treatment	No treatment
16. Quantification	No	No	No	No
**Score's total calculated**	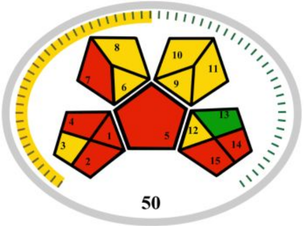	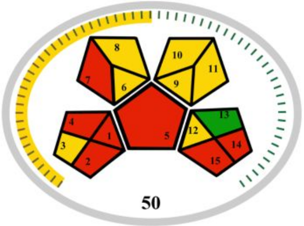	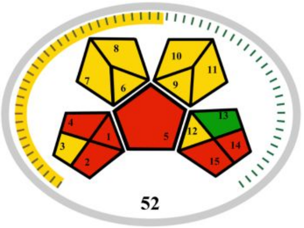	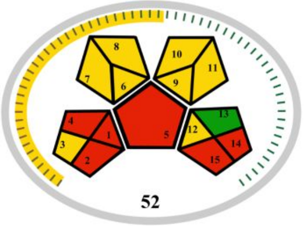

***According to NFPA. 704: Standard system for the identification of the hazards of materials for emergency response**. National Fire Protection Association: Quincy, MA, USA, 2022.

Table S2: Results obtained from the application of the AGREE tool for traditional (TBM), traditional with modifications (PMT), green (GBM) and green with modifications (PMG) methods.

Criteria	Extraction's methods								Weight
	TBM		PMT		GBM		PMG		
	Data	Score	Data	Score	Data	Score	Data	Score	
1. Sample treatment	Off-line	0.48	Off-line	0.48	Off-line	0.48	Off-line	0.48	1
2. Sample amount	0.1 g	0.98	0.1 g	0.98	0.1 g	0.98	0.1 g	0.98	1
3. Device positioning	Off-line	0.00	Off-line	0.00	Off-line	0.00	Off-line	0.00	1
4. Sample prep. stages	7 steps	0.20	4 steps	0.80	7 steps	0.20	4 steps	0.80	3
5. Automatization and miniaturization	Semi-auto/miniat.	0.75	Semi-auto/miniat.	0.75	Semi-auto/miniat.	0.75	Semi-auto/miniat.	0.75	1
6. Derivatization	No	1.00	No	1.00	No	1.00	No	1.00	3
7. Waste	91 mL	0.09	60.5 mL	0.14	91 mL	0.09	60.5 mL	0.14	3
8. Analysis throughput	30/30	1.00	30/30	1.00	30/30	1.00	30/30	1.00	1
9. Energy consumption	GC-MS/1.5	0.00	GC-MS//1.5	0.00	GC-MS/1.5	0.00	GC-MS/1.5	0.00	1
10. Source reagents	Some solvents	0.50	Some solvents	0.50	All solvents	1.00	All solvents	1.00	3
11. Toxicity	Yes, 58 mL	0.00	Yes, 27.5 mL	0.07	No	1.00	No	1.00	3
12. Operator's satefy	Toxic to aquatic life/highly flammable	0.60	Toxic to aquatic life/highly flammable	0.60	Highly flammable	0.80	Highly flammable	0.80	3
**Score's total calculated**	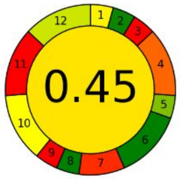		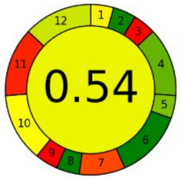		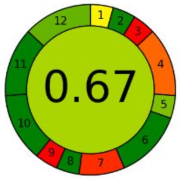		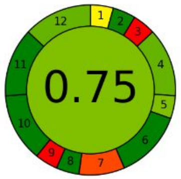		

.

Table S3: Results obtained from the application of the AGREEprep tool for traditional (TBM), traditional with modifications (PMT), green (GBM) and green with modifications (PMG) methods.

Criteria	Extraction's methods								Weight
	TBM		PMT		GBM		PMG		
	Data	Score	Data	Score	Data	Score	Data	Score	
1. Sample preparation placement	Ex-situ	0	Ex-situ	0	Ex-situ	0	Ex-situ	0	1
2. Hazardous materials	>20 mL	0	>20 mL	0	0	1	0	1	3
3. Sustainability, renewability, and reusability of materials	<25% of reagents/reagents	0	<25% of reagents/reagents	0	Only solvents/reagents	1	Only solvents/reagents	1	3
4. Waste	91 mL	0	60.5 mL	0	91 mL	0	60.5 mL	0	3
5. Size economy of the sample	0.1 g	1	0.1 g	1	0.1 g	1	0.1 g	1	1
6. Sample throughput	2	0.16	2	0.16	2	0.16	2	0.16	1
7. Integration and automation	>6 steps; semi-auto by voxtex	0	4 steps; semi-auto by voxtex	0.25	>6 steps; semi-auto by voxtex	0	4 steps; semi-auto by voxtex	0.25	3
8. Energy consumption	25 W	0.76	25 W	0.76	25 W	0.76	25 W	0.76	1
9. Post-sample preparation configuration for analysis	GC-MS	0.25	GC-MS	0.25	GC-MS	0.25	GC-MS	0.25	1
10. Operator's safety	4	0	3	0.25	2	0.5	1	0.75	3
**Score's total calculated**	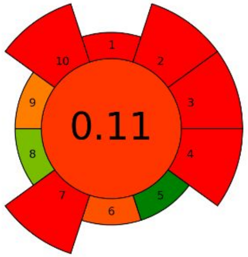		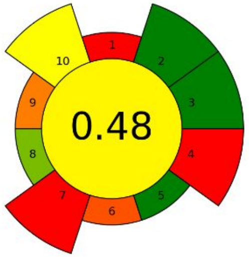		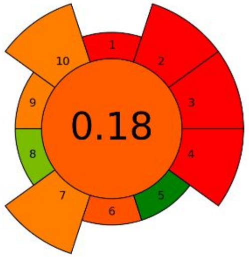		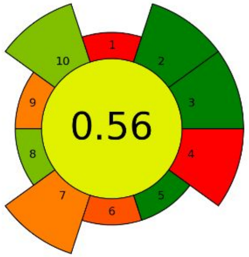		

Table S4: Yield of *Worsleya procera* leaf extracts and alkaloid-enriched fractions.

Solvents	Samples	Extraction yield (%)	
		TMM	TBM
Hexane and/or methanol (8:2 v/v)	EHEX	2.91 ± 0.02^{ns}	2.11 ± 0.12^{ns}
	EMeOH	35.42 ± 0.21^{ns}	25.80 ± 0.81^{ns}
		GMM	GBM
Heptane and/or ethanol (7:3 v/v)	EHEP	3.12 ± 0.01^{ns}	2.29 ± 0.04^{ns}
	EEtOH	37.60 ± 0.14^{ns}	27.07 ± 0.64^{ns}

Extracts	Patitions of solvents		
	Hexane	Heptane	EtOAc
EMeOH	0.96 ± 0.11^{ns}	-	4.94 ± 0.20*
	-	-	5.84 ± 0.14**
EEtOH	-	0.99 ± 0.21^{ns}	4.38 ± 0.15*
	-	-	5.16 ± 0.32**

Legend: Yields are expressed as mean ± standard error of the mean (n = 3). Statistical differences were evaluated by one-way ANOVA followed by Bonferroni's multiple comparison test. ns = not significant, (p > 0.05),* p< 0.05, ** p < 0.01, **** p < 0.0001.

Table S5: Annotation of alkaloids in *Worsleya procera* leaves by GC-MS and their confidence levels.

ID	Alkaloids	LRI exp.	LRI teoric	ΔRI (exp.–teor.)	NIST SI (%)	Confidence*
1	11,12-Dehydrolycorene	2349.8	2360	10.2	86	High
2	11-Deoxytazettine	2504.8	2485	19.8	96	High
3	3-Epimacronine	2804.6	2810	5.4	88	High
4	3-O-acetylpowelline	2792.8	2768.7	24.1	91	Moderate
5	3-*O*-Demetyl-hyppeastidine	2673.4	-	-	86	-
6	5,6-Dihydrobicolorine	2328.4	2321	7.4	87	High
7	Albomaculine	2797.5	2815	17.5	93	High
8	Galantamine	2391	2405	14	91	High
9	Galanthine	2690.2	2704	13.8	97	High
10	Galanthindole	2492	2487	5	98	High
11	Ismine	2266	2280	14	89	High
12	Lycorine	2748.2	2746	2.2	95	High
13	Macronine	2658.6	2689	30.4	93	Moderate
14	Tazettamide	2900.7	2866	34.7	93	Moderate
15	Tazettine	2642.4	2651	8.6	94	High

Legend: (*) The level of confidence assigned to each alkaloid annotation was based on two main parameters: (i) the deviation between the experimental linear retention index (LRI exp.) and the literature value (LRI teoric), and (ii) the similarity score obtained from spectral matching against the NIST library. Annotations were classified as high confidence when ΔRI was ≤ 20 units and the similarity score ≥ 85%. Moderate confidence was assigned when ΔRI was ≤ 40 units and the similarity score ≥ 80%. Cases falling outside these thresholds were considered low confidence, reflecting the intrinsic limitations of GC-MS as a unit-resolution technique.

Table S6: Relative intensities of compounds in extracts and different fractions of Worsleya procera leaves by GC-MS.

Nº	Alkaloids	LRI[a]	[M+]	EMeOH	FMHEX	FMEtOAc	EEtOH	FEHEP	FEEtOAc	EMEtOAc	EEEtOAc	References
				TIC relative area (%)								
**1**	11,12-Dehydrolycorene	2649.8	281 (44)	-	-	0.43	-	-	-	-	-	[58]
**2**	11-Deoxytazettine	2534.8	316 (2)	-	-	0.44	-	-	0.47	-	-	[59, 60]
**3**	3-Epimacronine	2804.6	329 (14)	-	-	-	-	0.90	1.46	-	2.63	[58, 61, 62] N
**4**	3-O-acetylpowelline	2892.8	344 (26)	-	1.06	0.20	-	-	-	-	-	[63]
**5**	3-*O*-Demetyl-hippeastidine	2673.4	389 (4)	-	-	0.50	-	-	-	-	-	[64]
**6**	5,6-Dihydrobicolorine	2328.4	239 (43)	-	-	0.32	-	-	-	-	-	[65]
**7**	Albomaculine	2930.3	345 (<1)	-	78.28	21.33	-	16.33	18.62	12.61	11.10	[6, 66, 67]
**8**	Galantamine	3111.6	289 (2)	0.40	1.76	1.04	0.31	0.83	1.04	1.16	1.22	[65, 68 - 70] N
**9**	Galanthine	2797.5	318 (3)	11.11	5.92	27.02	10.57	0.78	14.06	33.89	26.78	[59, 67, 71] N
**10**	Galanthindole	2391.0	281 (100)	1.84	-	3.25	1.41	-	0.47	-	-	[6, 58, 68 - 70, 72] N
**11**	Ismine	2690.2	257 (32)	2.22	-	0.72	1.73	-	0.78	-	-	[6, 59, 66, 67] N
**12**	Lycorine	2492.0	287 (23)	-	-	4.51	-	-	-	8.31	-	[6, 62, 66, 67, 71] N
**13**	Macronine	2450.9	355 (<1)	-	-	-	-	-	-	0.96	1.98	[61, 71]
**14**	Tazettamide	2266.0	341 (<1)	-	-	-	-	-	-	0.36	-	[74] N
**15**	Tazettine	2748.2	331 (<1)	1.37	-	14.67	1.19	0.78	15.16	36.46	43.75	[58, 59, 71] N
	**Others alkaloid subtypes detected***											
**16**	Unidentified type 1	2808.9	341 (<1)	-	-	-	-	-	-	1.06	2.08	[74]
**17**	Homolycorine type	2879.2	296 (2)	-	-	0.13	-	-	-	-	-	[74]
**18**	A1	2741.3	282 (<1)	-	11.29	-	-	3.27	-	-	-	[75]
**19**	Lycorine type	2741.9	288 (1)	1.99	-	-	1.68	-	3.87	-	-	[71]
**20**	Nerinine (I) type	2744.7	341 (<1)	-	-	3.11	-	-	-	-	6.41	[72]
**21**	Nerinine (II) type	2798.9	344 (<1)	9.11	-	13.78	9.23	-	-	-	-	[72]

**22**	Tazettine (I) type	2850.7	313 (30)	-	-	-	-	-	0.38	-	-	[59]
**23**	Tazettine (II) type	2903.0	355 (<1)	-	-	0.15	-	-	-	-	-	[59]
**24**	A13	2930.3	295 (90)	-	-	0.61	-	-	1.90	0.86	1.33	[71]
**25**	A4	3111.6	355 (2)	-	-	0.27	-	-	-	-	-	[75]
**26**	GG-1	2450.9	299 (7)	-	-	2.98	-	-	0.59	-	-	[69]
**24**	M+ 269	2219.8	269 (38)	-	-	-	-	-	0.61	-	-	[61]

Legend: [a]LRI: Linear Retention Index experimental; TIC: Total Ion Chromatogram.
